# Influence of the Composition of Cationic Liposomes on the Performance of Cargo Immunostimulatory RNA

**DOI:** 10.3390/pharmaceutics15092184

**Published:** 2023-08-23

**Authors:** Ali Bishani, Darya M. Makarova, Elena V. Shmendel, Mikhail A. Maslov, Aleksandra V. Sen‘kova, Innokenty A. Savin, Daniil V. Gladkikh, Marina A. Zenkova, Elena L. Chernolovskaya

**Affiliations:** 1 Institute of Chemical Biology and Fundamental Medicine SB RAS, Lavrentieva Ave. 8, 630090 Novosibirsk, Russia; ali1bishani@gmail.com (A.B.); senkova_av@niboch.nsc.ru (A.V.S.); savin_ia@niboch.nsc.ru (I.A.S.); medulla35@gmail.com (D.V.G.); marzen@niboch.nsc.ru (M.A.Z.); 2 Lomonosov Institute of Fine Chemical Technologies, MIREA—Russian Technological University, Vernadsky Ave. 86, 119571 Moscow, Russia; das.kozl@yandex.ru (D.M.M.); shmeldel@mirea.ru (E.V.S.); maslov_m@mirea.ru (M.A.M.)

**Keywords:** immunostimulatory RNA, cationic liposomes, lipoconjugates, cytokine-inducing activity, antitumor activity, IFN alpha

## Abstract

In this study, the impact of different delivery systems on the cytokine-inducing, antiproliferative, and antitumor activities of short immunostimulatory double-stranded RNA (isRNA) was investigated. The delivery systems, consisting of the polycationic amphiphile 1,26-bis(cholest-5-en-3-yloxycarbonylamino)-7,11,16,20 tetraazahexacosan tetrahydrochloride (2X3), and the lipid-helper dioleoylphosphatidylethanolamine (DOPE), were equipped with polyethylene glycol lipoconjugates differing in molecular weight and structure. The main findings of this work are as follows: (i) significant activation of MCP-1 and INF-α, β, and γ production in CBA mice occurs under the action of isRNA complexes with liposomes containing lipoconjugates with long PEG chains, while activation of MCP-1 and INF-γ, but not INF-α or β, was observed under the action of isRNA lipoplexes containing lipoconjugates with short PEG chains; (ii) a pronounced antiproliferative effect on B16 melanoma cells in vitro, as well as an antitumor and hepatoprotective effect in vivo, was induced by isRNA pre-complexes with non-pegylated liposomes, while complexes containing lipoconjugates with long-chain liposomes were inactive; (iii) the antitumor activity of isRNA correlated with the efficiency of its accumulation in the cells and did not explicitly depend on the activation of cytokine and interferon production. Thus, the structure of the delivery system plays a vital role in determining the response to isRNA and allows for the choice of a delivery system depending on the desired effect.

## 1. Introduction

Oncological diseases are among the most dreadful threats to human health globally and one of the greatest challenges of modern medicine. Conventional cancer treatments include surgical tumor removal with subsequent radiotherapy or chemotherapy [1]. These treatments are accompanied by such side effects as anemia, fatigue, inflammation, etc., due to their toxicity [2]. Moreover, the incomplete elimination of the tumor cells might cause a recurrence of the disease [3]. Thus, the development of new approaches for antitumor therapy is urgently needed. Immunotherapy has emerged as a groundbreaking treatment strategy against neoplastic diseases [4], as it helps the host immune system to eliminate tumor cells as well as prevent their recurrence [5]. At present, several immunotherapeutic approaches have exhibited remarkable results in clinical trials against different types of tumors, including specific immune therapies such as checkpoint blockades (ICBs) [6], adoptive T-cell therapy (CAR-T) [7], and therapeutic nucleic acids (TNAs), which have non-specific immunostimulation activity [8].

TNAs are various natural and chemically modified DNA or RNA molecules which are able not only to regulate the expression of target genes or direct the synthesis of the necessary protein, but also to harness the host immune system in antitumor therapy [9]. TNAs are recognized by different receptors in the cell known as pattern recognition receptors (PRRs) [10], which are able of detecting evolutionary conserved structures on the invading pathogens known as pathogen associated molecular patterns (PAMPs) [11] or molecules derived from host cells (DAMPs) including tumor cells, apoptotic cells, or products released from cells in response to such signals as hypoxia [12]. Receptors that detect nucleic acids are located in different cellular compartments: some of them are present in the cytosol (retinoic acid inducible gene-I (RIG-I)-like receptor, melanoma differentiation-associated protein 5 (MDA5), stimulator of interferon genes (STING), and less-studied NOD-like receptors (NLRP3, NOD2) [13]). Other receptors are located in the endosomes (Toll-like receptors 7\8, TLR 7/8) or cellular membrane (TLR3) [14]. The ability of nucleic acids to activate these receptors depends on their length, structure, localization, and sequence [15,16]. Recognition by such receptors leads to the activation of various signaling pathways, which can induce apoptosis and cause proliferation blockage and secretion of interferons and/or pro- or anti-inflammatory cytokines, depending on the nature of the ligand, activated receptor, and cell type [15,17]. Immunostimulatory TNAs can affect the tumor directly, by inhibiting proliferation and inducing differentiation or apoptosis, or indirectly, by activating the immune system for tumor elimination.

TNA action depends to a large extent on the efficiency of their delivery to the target cells. The most commonly used TNAs are conjugated with transport ligands [18] or are included in complexes with various cationic lipids, liposomes, polymers, or nanoparticles [19]. Numerous studies have been devoted to optimizing lipid formulations for the delivery of antisense oligonucleotides, siRNAs, mRNAs, and therapeutic vaccines in various modes of administration [20]. However, the delivery of immunostimulatory TNAs and the effect of delivery systems on the potency of both immunostimulation properties and its targeted biological effects have not yet been systematically studied.

Previously, a short 19-bp dsRNA with 3′-trinucleotide overhangs (immunostimulatory RNA, isRNA) molecule that exhibited pronounced antiproliferative activity with respect to cancer cells and immunostimulatory activity via activation of cytokine synthesis (type-I IFNs) was described by our group [21].

In this study, the impact of different delivery systems on the cytokine-inducing, antiproliferative, and antitumor activities of isRNA was investigated. The delivery systems consist of the polycationic amphiphile 1,26-bis(cholest-5-en-3-yloxycarbonylamino)-7,11,16,20 tetraazahexacosan tetrahydrochloride (2X3) and the lipid-helper dioleoylphosphatidylethanolamine (DOPE) (liposomes 2X3-DOPE), which were proven to effectively deliver various nucleic acids both in vitro and in vivo [22,23]. PEG-containing lipoconjugates with a loop-like structure have recently been synthesized, and we showed that the equipment of the liposomes with compounds having long oligoethylene hydrophilic spacers increases the efficiency of the TNAs delivery into cells as compared to Lipofectamine 3000 [24]. Here we describe the synthesis of several novel polyethylene glycol (PEG)-containing loop-like lipoconjugates differing in their molecular weight of PEG (800, 1500 and 2000 Da) and provide a detailed comparison of liposomes containing these lipoconjugates with PEG- and folate-lipoconjugates previously described [25,26] in liposome-mediated isRNA delivery in vitro and in vivo.

## 2. Materials and Methods

### 2.1. Synthesis of diP800, diP1500, diP2000

#### 2.1.1. Synthesis of diP800

Solutions of *rac*-1-*O*-(4-nitrophenyloxycarbonyl)-2,3-di-*O*-tetradecylglycerol (pNDg) (239.8 mg, 0.369 mmol) and Et_3_N (0.05 mL, 0.369 mmol) in anhydrous CH_2_Cl_2_ (10 mL) were added to a solution of diamine (110.2 mg, 0.123 mmol) in anhydrous CH_2_Cl_2_ (10 mL). The reaction mixture was stirred for 20 h at 24 °C, washed with 3% aq. HCl (1 × 25 mL), saturated with aq. NaCl (4 × 25 mL) up to pH 7, dried with Na_2_SO_4_, and filtered, followed by evaporation of the solvent in vacuum. The product was isolated via column chromatography using chloroform-methanol (50:1 vol.) as an eluent. The compound diP800 was obtained at a 70% yield (164.3 mg) in the form of a colorless crystallized oil. ^1^H NMR (300 MHz, CDCl_3_): 0.86 (t, *J* = 6.7 Hz, 12 H, 4 CH_3_); 1.15–1.40 (m, 88 H, 4 (CH_2_)_11_); 1.43–1.69 (m, 8 H, 4 OCH_2_CH_2_); 3.28–3.37 (m, 4 H, 2 NHCH_2_); 3.39–3.89 (m, 90 H, 42 OCH_2_, 2 OCHCH_2_); 4.08 (dd, *J* = 5.4 Hz, *J* = 11.4 Hz, 2 H) and 4.18 (dd, *J* = 4.4 Hz, *J* = 11.4 Hz, 2 H, 2 CH_2_OC(O)); 5.00–5.40 (m, 2 H, 2 NH). ^13^C NMR (60 MHz, CDCl_3_): 14.08, 22.66, 26.04, 26.10, 29.33, 29.49, 29.61, 29.63, 29.67, 30.02, 31.67, 31.90, 32.13, 64.34, 69.32, 70.48, 70.53, 70.58, 71.11, 71.60, 71.76, 156.41.

The compounds diP1500 and diP2000 were synthesized as described for diP800 (the amounts of diamines and pNDg, yields, and physicochemical characteristics are given).

#### 2.1.2. Synthesis of diP1500

diP1500 was synthesized from diamine (60 mg, 0.04 mmol) and pNDg (78 mg, 0.12 mmol) and was obtained at a 58% yield (61.2 mg) in the form of a colorless crystallized oil. The ^1^H NMR spectrum (300 MHz, CDCl_3_): 0.87 (t, *J* = 6.5 Hz, 12 H, 4 CH_3_); 1.25–1.55 (m, 88 H, 4 (CH_2_)_11_); 1.43–1.69 (m, 8 H, 4 OCH_2_CH_2_); 1.71–1.89 (m, 4 H, 2 NHCH_2_CH_2_); 3.27–3.44 (m, 4 H, 2 NHCH_2_CH_2_); 3.64–3.89 (m, 150 H, CH_2_(OCH_2_CH_2_)_33_OCH_2_, 4 OCH_2_CH_2_, 2 OCH_2_CHO); 4.11 (dd, *J* = 5.4 Hz, *J* = 11.4 Hz, 2 H) and 4.24 (dd, *J* = 4.4 Hz, *J* = 11.4 Hz, 2 H, 2 CH_2_OC(O)); 5.21–5.40 (m, 2 H, 2 NH). The ^13^C NMR spectrum (60 MHz, CDCl_3_): 14.08, 22.43, 22.66, 25.79, 29.33, 29.49, 29.61, 29.63, 31.67, 40.91, 69.32, 70.07, 71.11, 156.43.

#### 2.1.3. Synthesis of diP2000

diP2000 was synthesized from diamine (80 mg, 0.04 mmol) and compound (pNDg) (78 mg, 0.12 mmol) as described for compound (diP800). The compound (diP2000) was obtained at a yield of 64% (76.9 mg) in the form of a colorless crystallized oil. The ^1^H NMR spectrum (300 MHz, CDCl_3_): 0.86 (t, *J* = 6.7 Hz, 12 H, 4 CH_3_); 1.15–1.55 (m, 88 H, 4 (CH_2_)_11_); 1.43–1.69 (m, 8 H, 4 OCH_2_CH_2_); 1.71–1.89 (m, 4 H, 2 NHCH_2_CH_2_); 3.27–3.44 (m, 4 H, 2 NHCH_2_CH_2_); 3.64–3.89 (m, 186 H, CH_2_(OCH_2_CH_2_)_42_OCH_2_, 4 OCH_2_CH_2_, 2 OCH_2_CHO); 4.11 (dd, *J* = 5.4 Hz, *J* = 11.4 Hz, 2 H) and 4.24 (dd, *J* = 4.4 Hz, *J* = 11.4 Hz, 2 H, 2 CH_2_OC(O)); 5.21–5.40 (m, 2 H, 2 NH). The ^13^C NMR spectrum (60 MHz, CDCl_3_): 14.08, 22.43, 22.66, 25.79, 29.33, 29.49, 29.61, 29.63, 31.67, 40.91, 69.32, 70.07, 71.11, 156.43.

### 2.2. Liposomes Preparation

**Figure 1:**
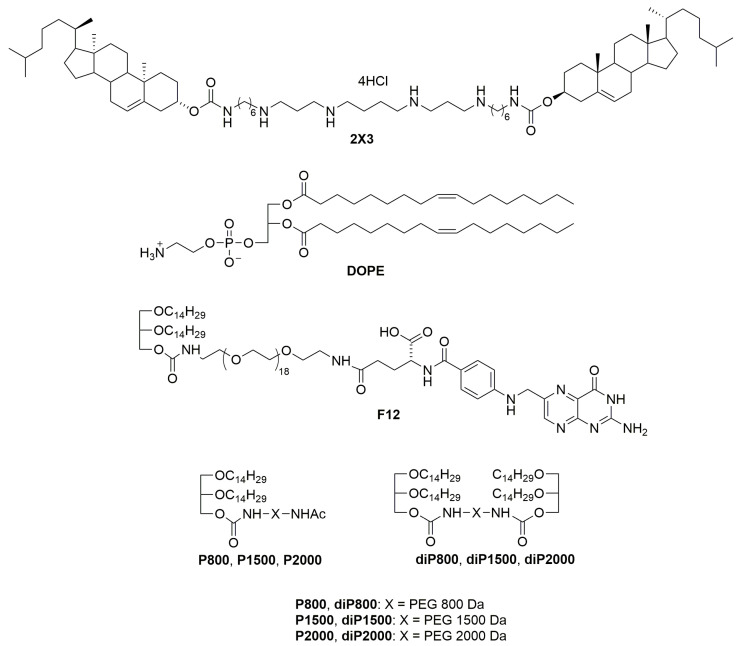
Structures of cholesterol-based polycationic amphiphile 1,26-bis(cholest-5-en-3_-yloxycarbonylamino)-7,11,16,20-tetraazahexacosan tetrahydrochloride (2X3), lipid helper dioleoylphosphatidylethanolamine (DOPE), lipoconjugates: *O*,*O*′[*rac*-2,3-di(tetradecyloxy) propyl-1-oxycarbonylamino] octadecaethylene glycol (P800), *O*,*O*′-bis[*rac*-2,3-di(tetradecyloxy) propyl-1-oxycarbonylamino] octadecaethylene glycol (diP800), *O*,*O*′[*rac*-2,3-di(tetradecyloxy) propyl-1-oxycarbonylamino] poly(ethylene glycol_1500_) (P1500), *O*,*O*′-bis[*rac*-2,3-di(tetradecyloxy) propyl-1-oxycarbonylamino] poly(ethylene glycol_1500_) (diP1500), *O*,*O*′[*rac*-2,3-di(tetradecyloxy) propyl-1-oxycarbonylamino] poly(ethylene glycol_2000_) (P2000), *O*,*O*′-bis[*rac*-2,3-di(tetradecyloxy) propyl-1-oxycarbonylamino] poly(ethylene glycol_2000_) (diP2000) and *O*-{2-[rac-2,3-di(tetradecyloxy) prop-1-yloxycarbonyl]aminoethyl}-*O*′-[2-(pteroyl-L-glutam-5yl)aminoethyl]octadecaethyleneglycol (F12) is used for liposome preparation.

All liposomal formulations were prepared via the hydrating thin lipid film method [27]. Briefly, a solution of the polycationic lipid 1,26-bis(cholest-5-en-3β-yloxycarbonylamino)-7,11,16,20-tetraazahexacosane tetrahydrochloride (2X3) [28] in a mixture of CHCl_3_-CH_3_OH (1:2 vol.) was added to a solution of 1,2-dioleoyl-*sn*-glycero-3-phosphoethanolamine (DOPE, Avanti Polar Lipids, Alabaster, AL, USA) in CHCl_3_ at a molar ratio of 1:1 and gently stirred. To obtain different liposomal formulations, a solution of one of the lipoconjugates (F12) [29], P800, P1500, P2000 [26], diP800, diP1500, diP2000, 2% or 4% mol.) (Figure 1) in CHCl_3_-CH_3_OH (1:1) was added to the 2X3-DOPE mixture, and organic solvents were removed in vacuo. The obtained lipid film was dried for 4 h at 0.1 Torr to remove residual organic solvents and was hydrated in deionized water (MilliQ, Burlington, MA, USA) at 4 °C overnight. The resulting liposomal dispersion was sonicated for 15 min at 70–75 °C in a bath-type sonicator (Bandelin Sonorex Digitec DT 52H, Berlin, Germany), flushed with argon, and stored at 4 °C. In the resulting dispersion, the cationic lipid 2X3 concentration was 1 mM.

### 2.3. Synthesis of isRNA

Oligoribonucleotide (strand 1: 5′-GUGUCAGGCUUUCAGAUUUUUU-3′, strand 2: 5′-AAAUCUGAAAGCCUGACACUUA-3′) synthesis was carried out as described previously [26]. isRNAs (50 µM) were annealed in a buffer containing 30 mM HEPES-KOH (pH 7.4), 100 mM sodium acetate, and 2 mM magnesium acetate via heating at 90 °C for 5 min, followed by cooling to room temperature. The siRNA preparations were stored at −20 °C until use.

### 2.4. isRNA/Liposome Complexes Preparation

The complexes of isRNA and liposomes were prepared at N/P ratios of 6/1 in serum-free OptiMEM medium (Invitrogen, Waltham, MA, USA) by mixing equal amounts of isRNA solution with a final concentration of 3.5 µM and liposome solution with a final concentration of 150 µM; then they were incubated at 24 °C for 20 min.

Particle size and zeta potential were measured using a Zetasizer Nano ZS (Malvern Panalytical Ltd., Malvern, UK). The average hydrodynamic diameter (nm) was obtained from particle number distributions; the measurements were repeated three times.

### 2.5. PBMC Cell Culture and isRNA Transfection

Venous blood was obtained from healthy donors at the E. Meshalkin National Medical Research Center of the Ministry of Health of the Russian Federation, Novosibirsk, Russia. Human peripheral blood mononuclear cells (PBMCs) were isolated using Ficoll Paque Plus (1.077 g/mL density; GE Healthcare, Chicago, IL, USA) according to the manufacturer’s instructions. An enriched monocyte population was isolated via plastic adherence. After a 4-h incubation at 37 °C in a 5% CO_2_/95% air atmosphere, non-adherent cells were removed via repeated gentle washing with the medium. The cells were replated in 24-well plates (0.5 × 10^5^ cells per well) in duplicates and left for 24 h in RPMI medium 1640 (Thermofisher, Waltham, MA, USA) supplemented with 10% fetal bovine serum. Then, the cells were transfected with isRNA (100 nM) using liposomes (6.3 µM) and incubated at 37 °C in a 5% CO_2_/95% air atmosphere. The medium was collected 6, 16, and 24 h after transfection and stored at −80 °C.

### 2.6. Analysis of Cytokine Levels in PBMC Culture Medium by ELISA

The levels of IFN-α, TNF-α, and IL-6 in the culture medium were measured with ELISA kits (Vector-Best, Novosibirsk, Russia). The analysis was performed according to the protocols provided by the manufacturer. Samples were measured in duplicate. Finally, the optical density was measured at a wavelength of 450 nm, using a Multiskan RC multichannel photometer (Labsystems, Vantaa, Finland).

### 2.7. Tumor Cell Lines

Melanoma B16 cells were obtained from the N. N. Blokhin Cancer Research Center, Moscow, Russia. B16 cells were grown in DMEM (Sigma-Aldrich, St. Louis, MO, USA) supplemented with 10% FBS (HyClone, GE Healthcare, Chicago, IL, USA) and 1% antibiotic–antimycotic solution (MP Biomedicals, Santa Ana, CA, USA). RLS40 drug-resistant murine lymphosarcoma was previously developed in our laboratory from lymphosarcoma LS susceptible to chemotherapy [26]. RLS40 cells were cultured in IMDM (Sigma-Aldrich, St. Louis, MO, USA) supplemented with 10% FBS, 1% antibiotic–antimycotic solution, and 40 nM vinblastine. All cells were cultivated in a humidified atmosphere containing 5% CO_2_/95% air at 37 °C and were regularly passaged to maintain the exponential growth.

### 2.8. Antiproliferative Activity In Vitro

Cells were seeded in 96-well flat-bottom plates at a density of 3 × 10^3^ for B16 and 1 × 10^3^ for RLS40 per well in antibiotic-free medium 1 day before transfection. Cells on six plates (each cell line) were transfected in triplicate with 100 nM isRNA complexed with liposomes 2X3-DOPE or P2000 at N/P = 6/1 or with liposomes alone and incubated at 37 °C. On a daily basis after transfection, 10 μL of a 0.5 mg/mL solution of water-soluble tetrazolium WST-1 (Roche, Basel, Switzerland) was added to each well of one plate, and cells were incubated for 30 min at 37 °C in a CO_2_ incubator. Then the absorbance was measured spectrophotometrically using Multiscan RC (Labsystems, Vantaa, Finland) at 450 and 620 nm.

### 2.9. Cellular Accumulation Assay

One day before the experiment, B16 or RLS40 cells in the exponential phase of growth were seeded on 48-well plates at a density of 4.5 × 10^4^ cells/well. Following a 24-h incubation, Cy5.5-labeled isRNA or its complexes with liposomes were added to the cells. Four hours after Cy5.5-isRNA addition, B16 cells were detached with TrypLE (Thermo Fisher Scientific, Waltham, MA, USA), and RLS40 cells were collected via centrifugation and fixed with 2% formaldehyde in phosphate-buffered saline (PBS). Cells were analyzed using a NovoCyte flow cytometer (ACEA Biosciences, San Diego, CA, USA); 8000 cells from each sample were analyzed. NovoExpress 1.1.0 (ACEA Biosciences, San Diego, CA, USA) was used for data analysis.

### 2.10. Real-Time Quantitative PCR Assay

Total RNA was isolated from B16 or RLS40 cells using TRIzol Reagent according to the manufacturer’s instructions. cDNAs were synthesized using RT buffer and M-MuLV-RH revertase (Biolabmix, Novosibirsk, Russia) according to the manufacturer’s instructions. qPCR was carried out using HS-qPCR (×2) master mix (Biolabmix, Novosibirsk, Russia) according to the manufacturer’s instructions. Real-time PCR was carried out using an CFX96 Real Time System (Bio-Rad Laboratories Inc., Hercules, CA, USA) according to the following scheme: one cycle—3 min, 95 °C; 40 cycles—30 s, 95 °C; 30 s, 58 °C; and 30 s, 72 °C. All measurements were carried out in triplicate. *Tbp* was used as the reference gene. The sequences of primers and probes for *Pkr* and *Tbp* are listed in Table 1. The relative level of gene expression was calculated using the Bio-Rad CFX software (Bio-Rad Laboratories Inc., Hercules, CA, USA).

### 2.11. Animals

Adult female CBA and C57BL\6 mice (20–24 g) were obtained from the Center for Genetic Resources of Laboratory Animals at the Institute of Cytology and Genetics SB RAS. Animals were kept in the vivarium of the Institute of Chemical Biology and Fundamental Medicine, SB RAS, with a natural light regime on a standard diet for laboratory animals (GOST (State Standard) 34566-2019 [30]) in compliance with the international recommendations of the European Convention for the Protection of Vertebrate Animals Used for Experimental Studies (1997) and the rules of laboratory practice in the performance of preclinical studies in the Russian State Standards (R 51000.3-96 and 51000.4-96). The experimental protocols were approved by the Committee on the Ethics of Animal Experiments with the Institute of Cytology and Genetics of SB RAS (protocol #22.11 from 30 May 2014).

### 2.12. Analysis of IFN-α Levels in Murine Blood Serum by ELISA

CBA mice (*n* = 3) were injected intravenously with 10 µg (750 nmol) isRNA precomplexed with 47.25 nmol of cationic liposomes at an N/P ratio of 6/1 or with liposomes only in 200 µL of sterile OptiMEM medium. The blood was collected after 6 h from the retroorbital sinus and used to prepare the serum via clot coagulation for 30 min at 37 °C and subsequent centrifugation. The levels of IFN-α were measured via ELISA using the Mouse interferon alpha 2 matched antibody pair kit (ab215409, Abcam, Cambridge, UK) in accordance with the manufacturer’s instructions. Individual serum samples were measured in duplicate.

### 2.13. Cytokine Profiling

A bead-based multiplex LEGENDplex^TM^ Mouse Inflammation Panel (13-plex) (Biolegend, San Diego, CA, USA) was used for cytokine and chemokine quantification in accordance with the manufacturer’s instructions. Individual serum samples obtained from CBA mice treated with isRNA/liposome were assayed in duplicate. Analysis was performed using the NovoCyte 3000 flow cytometer (ACEA Bioscience, San Diego, CA, USA). The data were analyzed using Legendplex online software and specified as pg/mL.

### 2.14. Antitumor Studies In Vivo

The antitumor effect of isRNA precomplexed with cationic liposomes in vivo was evaluated using lymphosarcoma RLS40 and melanoma B16 cells. Tumors were initiated in CBA mice via intramuscular implantation of 10^6^ RLS40 cells in 0.1 mL of sterile saline buffer into the right thigh. In the case of melanoma, B16 tumors were initiated in C57BL\6 mice via subcutaneous implantation of 10^5^ B16 cells in 0.1 mL of sterile saline buffer.

After transplantation, the mice were randomly assigned to 5 groups based on further treatment (*n* = 5 mice per group): (1) control group, mice were injected with OptiMEM (200 μL per animal); (2) and (3) mice injected with cationic liposomes (2X3-DOPE or P2000) in 200 μL of sterile OptiMEM per animal; (4) and (5) mice injected with isRNA/liposomes (2X3-DOPE or P2000) in 200 μL of sterile OptiMEM per animal. The complexes were prepared as described above (10 μg of isRNA per mouse). In total, three injections were made at 3-day intervals starting from day 10 after tumor implantation (day 0). The tumor volumes were measured every 2 days using calipers in an investigator-blinded fashion. Tumor volumes were calculated as V = π/6 × length × width × height. On day 21, the animals were euthanized, then tumors, livers, and spleens were collected and fixed in 10% neutral-buffered formalin for further histological analysis.

### 2.15. Histology

For histological studies, murine livers and spleens fixed in 10% neutral-buffered formalin (BioVitrum, Moscow, Russia) were embedded in HISTOMIX paraffin (BioVitrum, Moscow, Russia). The paraffin sections (5 μm) were sliced on a Microm HM 355S microtome (Thermo Fisher Scientific, Waltham, MA, USA) and stained with hematoxylin and eosin. All images were obtained using an Axiostar Plus microscope equipped with an Axiocam MRc5 digital camera (Zeiss, Oberkochen, Germany) at ×400 magnification for the liver and ×100 for the spleen.

Morphometric analysis was performed using a counting grid consisting of 100 testing points in a testing area equal to 3.2 × 10^6^ μm^2^. Spleen analysis included evaluation of volume densities (Vv, %) of white and red pulps. Liver analysis included evaluation of the volume densities (Vv, %) of normal hepatocytes, dystrophy, and necrosis as well as the numerical density (Nv) of binuclear hepatocytes in the liver parenchyma from 10 random fields in each sample, forming 50 random fields for each group of mice in total.

### 2.16. Ethical Statements

Human peripheral blood mononuclear cells (PBMCs) were isolated from healthy donors, and all donors provided informed consent. All animal procedures were conducted in strict accordance with the recommendations for the proper use and care of laboratory animals (ECC Directive 86/609/EEC). The protocol was approved by the Committee on the Ethics of Animal Experiments of the Administration of the Siberian Branch of the Russian Academy of Sciences.

### 2.17. Statistical Analysis

GraphPad Prism 8.4.3 was used to perform statistical analysis (GraphPad Software, Inc., San Diego, CA, USA), data is represented by the mean ± standard deviation (SD). Statistically significant differences were determined using an ordinary two-way ANOVA with Dunnett’s multiple comparisons test. Differences were considered statistically significant at a *p* value of 0.05.

## 3. Results

We prepared a set of cationic liposomes based on the previously described 2X3-DOPE core system for the delivery of isRNA (Table 2, Figure 1). The ratio between 2X3 and DOPE was changed to 1:1, which is different from the value of 1:2 used earlier, since, according to the literature, this ratio (1:1) contributes to the more efficient performance of liposomes [23]. isRNA and liposomes form complexes due to electrostatic interactions between negatively charged phosphate groups of isRNA and four positively charged protonated amino groups in 2X3. Liposome surface modification with polyethylene glycol and folate lipoconjugate was used to stabilize liposomes and their complexes with isRNA in the bloodstream and ensure its effective accumulation in the target cells, as well as to reduce the rate of excretion of complexes and improve the bioavailability of the cargo. For this purpose, PEG-containing lipoconjugates differing in molecular weight (800, 1500, and 2000 Da) and structure were used. The P-series contains a PEG attached to 1,2-di-*O*-ditetradecyl-*rac*-glycerol as an anchor, and the diP-series is represented by a PEG chain of different lengths containing similar anchor groups at both ends. The lipoconjugates of the diP-series, when included in the composition of liposomes, are anchored on the surface of the liposomes by both anchor groups and adopt a loop-like structure, which allows for the formation of a denser but thinner protective shell. The content of lipoconjugates in the liposomal formulation varied from 2 to 4% to ensure effective protection from aggregation in serum and opsonization by serum proteins. Finally, a folate-containing lipoconjugate (FC) built of 1,2-di-*O*-ditetradecyl-*rac*-glycerol and folic acid connected via an 800 Da PEG spacer has been added to liposomes alone or together with PEG-containing lipoconjugates in order to facilitate the interaction of liposomes with folate receptors, which are expressed on several types of tumor cells [25]. The composition and some characteristics of the prepared liposomes and lipoplexes are listed in Table 2. It can be seen that the sizes of the initial liposomes (2X3-DOPE) do not exceed 130 nm. The addition of lipoconjugates to 2X3-DOPE composition led to a 2-fold reduction in their sizes for the P- and F-series but did not change the size of diP-liposomes. In Table 2, the characteristics of isRNA/liposome complexes prepared at an N/P of 6/1 are displayed; this ratio was previously determined to be optimal for siRNA delivery in terms of a balance of efficiency and absence of toxicity [22]. The data show that the formation of lipoplexes with isRNA somewhat increases the size of the particles, but they remain in the acceptable range for the delivery of TNAs.

### 3.1. Activation of the Cytokines Production by isRNA/Liposomes in Human PBMCs

The immunostimulatory effects of selected liposomes and isRNA/liposome complexes were investigated in primary cultures of human PBMS in order to evaluate the possible therapeutic potential of delivery systems and compare with the results of in vivo experiments. We measured the level of IFN-α since it has antitumor and anti-viral activity and plays a vital role in immunotherapy; additionally, it was of great importance to monitor the levels of pro-inflammatory cytokines, especially IL-6 and TNF-α, to avoid acute inflammation; accordingly, these specific cytokines were screened for. Human PBMC cell culture was established; the cells were seeded in 24-well plates and incubated with empty liposomes or isRNA lipoplexes. The medium from the cell culture was collected at 6, 16, and 24 h post-transfection. ELISA was used to measure the levels of IFN-α for evaluation of its anti-tumor potential. TNF-α and IL-6 levels were measured via the same method to assess toxicity associated with possible inflammation side effects.

**Figure 2:**
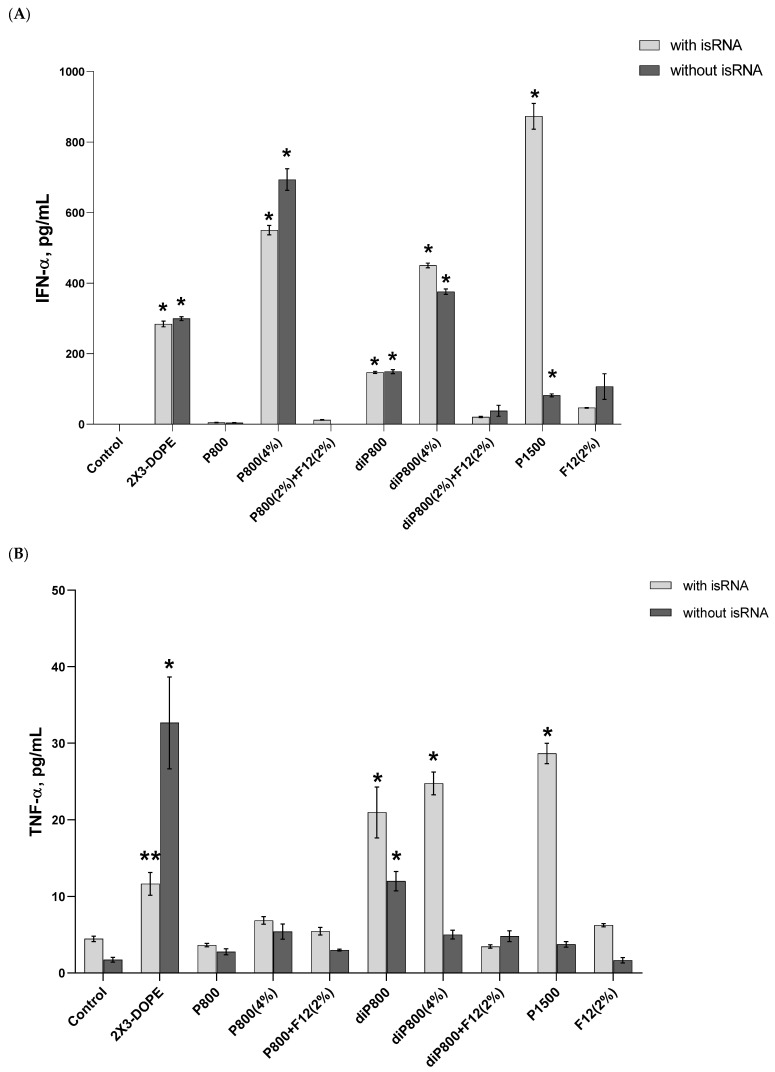

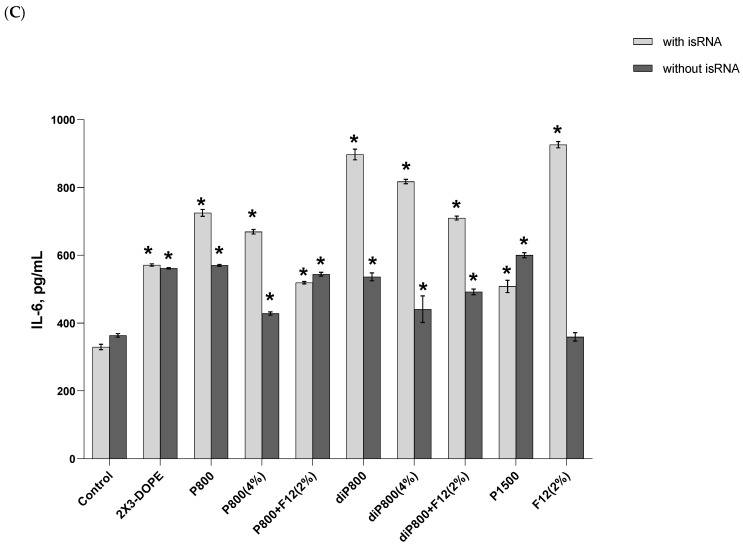
Cytokine levels in human PBMC primary cell culture after cell transfection with isRNA/liposomes complexes. The levels of IFN-α (**A**) and TNF-α (**B**) 16 h after transfection and the levels IL-6 (**C**) 6 h after transfection were measured via ELISA. The data represent mean ± standard deviation (SD). Statistically significant differences between samples treated with isRNA/liposomes and the samples treated with empty liposomes are indicated by asterisks (* *p* < 0.001, ** *p* < 0.005); ordinary two-way ANOVA, Dunnett’s multiple comparisons test. Data for other time points are presented in Supplementary Data (Figure S1).

Results reveal that no significant changes in the levels of IFN-α were observed 6 h after transfection (Figure S1); then the levels significantly increased at the 16-h time point (Figure 2A) and dropped again to their initial values at 24 h. The isRNA/P1500 complex induced the highest rise in the levels of IFN-α 16 h after transfection (872.93 pg/mL); at the same time, P1500 liposomes themselves practically do not affect the level of IFN-α. In the case of other lipoplexes, the effects of isRNA/liposome complexes and empty liposomes were similar, which indicates that these liposomes have an immunostimulatory effect and do not contribute to the manifestation of the specific activity of isRNA in this model. The inclusion of a folate-containing lipoconjugate in the composition almost completely blocked the desired effect (Figure 2A and Figure S1A).

A slight increase in the level of TNF-α (up to 33 pg/mL) was detected at the 16-h time point; this increase was induced by the 2X3-DOPE core system and isRNA complexes with diP800 (2 and 4%) and P1500, and these liposomes themselves did not cause such an effect. A moderate rise in the level of IL-6 was observed 6 h after transfection; higher levels were observed for isRNA complexes with diP800 and F12 (Figure 2C). The levels of IL-6 in cells transfected with P1500 and isRNA/P1500 are slightly elevated and do not differ significantly from the levels in untreated cells (Figure 2C). diP800 (2%) and isRNA/diP800 (2%) induced an approximately 3-fold increase in the levels of IL-6. Given the observed results, the isRNA/P1500 formulation proved to be the most favorable since it did not cause a pronounced activation of pro-inflammatory cytokine secretion, providing specific and efficient IFN-α production (Figure 2A).

### 3.2. Activation of the Cytokine Production Induced by isRNA/Liposomes in Mice

**Figure 3:**
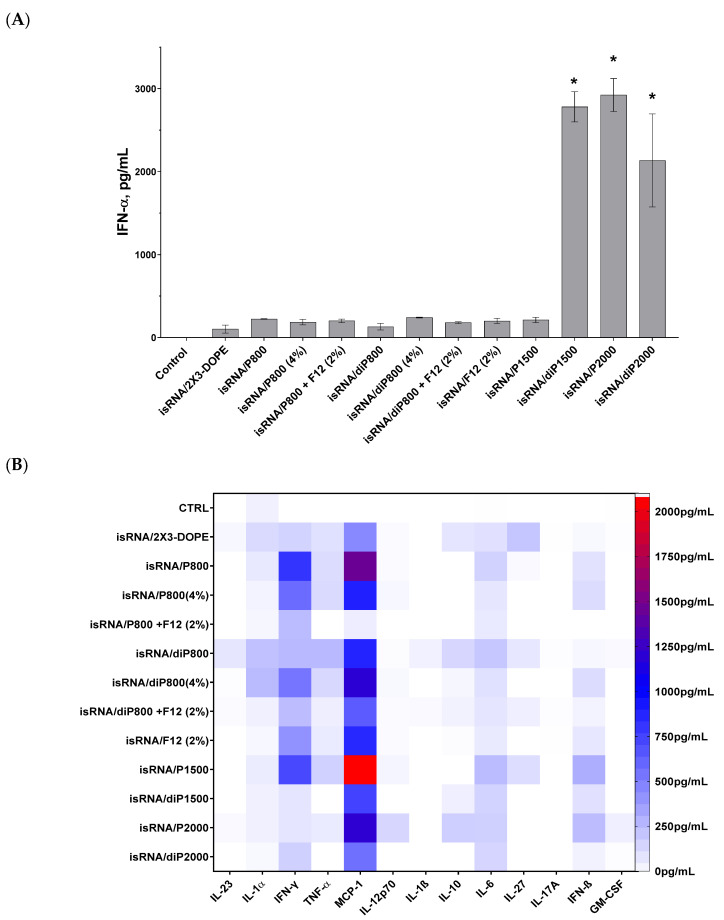
Cytokine production in CBA mice (*n* = 3) 6 h after i.v. administration of isRNA/liposomes complexes. (**A**) Serum IFN-α levels measured via ELISA. The data represent mean ± standard deviation (SD) calculated from samples from three mice measured in duplicates. Statistically significant differences between experimental groups and the control group are indicated by asterisks (* *p* < 0.001); ordinary two-way ANOVA, Dunnett’s multiple comparisons test. (**B**) Cytokine profiling measured using LEGEND plex™ Mouse Inflammation Panel (13-plex) kit. Numerical data are presented in Supplementary Data Table S1.

In order to evaluate the effect of liposomal compositions on the immunostimulatory activity of isRNA in vivo, the activity of lipoplexes consisting of 10 μg of isRNA and various liposomes at a concentration corresponding to N/P = 6/1 was assessed by measuring the levels of inflammation-related cytokines and interferons in the blood serum of CBA mice 6 h after intravenous (i.v.) administration of the lipoplexes. Profiling of 13 anti-inflammatory and pro-inflammatory cytokines was carried out using the LEGEND plex™ Mouse Inflammation Panel (13-plex) kit as described in the Section 2; the level of IFN-α not included in the kit was measured using ELISA (Figure 3). Taking into account the positive effect of a lipoconjugate with a long PEG chain on the bioperformance of the delivered isRNA, we added P2000, as well as diP1500 and diP2000, to the line of investigated liposomes.

The results indicate that the main cytokines that were activated in response to isRNA/liposome administration are monocyte chemoattractant protein 1 (MCP-1) and interferons α and γ. Moderate activation of interferon β and pro-inflammatory cytokines IL-6 and, to a lesser degree, IL-1α and TNFα, as well as a slight activation of anti-inflammatory IL-10 and IL-27, were also observed. The levels of IL-23, IL-12p70, IL-β, IL-17A, and GM-CSF were not noticeably changed in response to isRNA administration.

The obtained data demonstrates that lipoplexes containing diP1500, P2000, and diP2000 induced the most significant rise in the levels of IFN-α in comparison to the control group and other groups receiving lipoplexes. The greatest rise was induced by complexes with P2000; this implies that increasing the length of the PEG chain subsequently improves the performance of isRNA in activating IFN-α secretion (Figure 3A). The higher levels of IFN-γ were observed after induction by isRNA complexes with P800 and P1500 (≈800 pg/mL), and increased levels of IFN-β were observed in mice treated with P1500- or P2000-containing complexes (≈300 pg/mL). Noticeable low-level activation of pro-inflammatory cytokines IL-6 and TNF-α (≈250 pg/mL) was detected in the groups treated with P1500 and diP800 complexes. The obtained data demonstrates that the core system (2X3-DOPE) caused a moderate increase in cytokines level when used without PEG-containing lipoconjugates.

Data analysis shows that elongation of the PEG chain in the liposome shield has a significant effect on the immune response to isRNA delivered by the liposome under consideration: lipoplexes with diP1500, P2000, and diP2000 induced a remarkable increase in the levels of IFN-α and γ without causing an extensive rise in the levels of pro-inflammatory cytokines (IL-6 and IL-10). The increase in the content of 800 Da PEG lipoconjugate in liposome formulation from 2% to 4% did not have a pronounced effect on the cytokine syntheses: it reduced the desired effect of the P-series and slightly increased the effect of the diP-series, which itself is less effective than the P-series (Figure 3B). In contrast to the data obtained in vitro, an increase in P800 lipoconjugate in the formulation did not significantly change the immunostimulatory effect of the respective lipoplexes in vivo. An inverse relationship is observed for diP800 liposomes, where an increase in the percentage of lipoconjugate in the composition increases the levels of secreted cytokines MCP-1, IFN-α, IFN-γ, and IFN-β. However, the levels of cytokines were not as significant as those induced by lipoplexes containing lipoconjugates with longer PEG chains. The inclusion of a folate-containing lipoconjugate in the composition blocks the positive effect of the increased amount of PEG-lipoconjugates (Figure 3A and Figure S1A)

Comparison of liposomes of the P-series (one anchor group on PEG lipoconjugate) with those of the diP-series (two anchor groups) shows that stronger anchoring of PEG and the reduction of the shield size weaken the immunostimulatory effect of isRNA/liposomes and reduce the activation of interferons and MCP-1 secretion. In the case of lipoconjugates with PEG 800 Da, diP800 provoked a larger increase in the levels of pro-inflammatory cytokines than P800. The incorporation of folic acid-containing lipoconjugate F12 into liposomal formulations alone or additionally to diP800 does not have a positive effect, but rather reduces immunostimulation. Finally, comparison of all tested formulations in terms of positive (interferons and MCP-1 production) and negative (pro-inflammatory activity) properties allows us to select P2000 for further in vivo experiments.

### 3.3. Antiproliferative Activity of isRNA/Liposomes In Vitro

**Figure 4:**
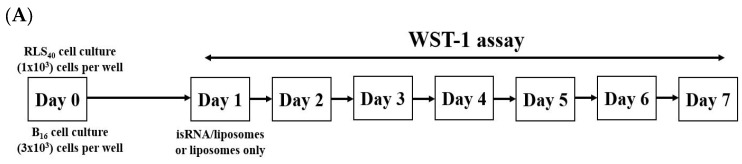

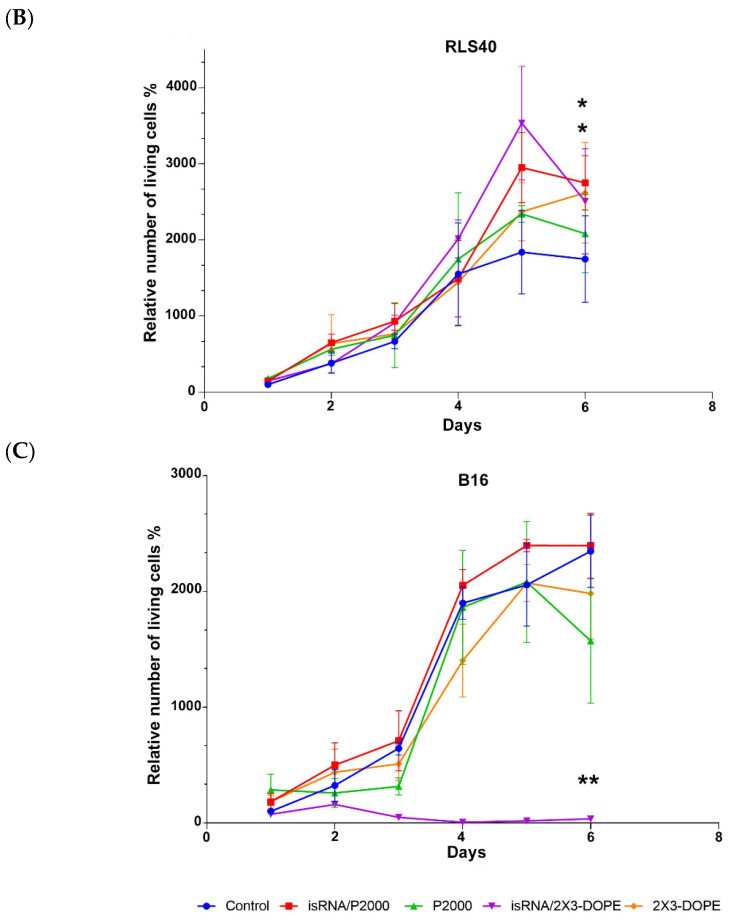
Antiproliferative activity of isRNA/liposomes on melanoma B16 and lymphosarcoma RLS40 tumor cells. (**A**) The experimental scheme. (**B**) Dynamics of lymphosarcoma RLS40 cell growth. (**C**) Dynamics of melanoma B16 cell growth. The relative number of living cells was assayed via WST-1; the number of cells at day 2 in control was set as 1. Statistically significant differences between experimental groups (isRNA/P2000 and P2000 in RLS40, isRNA/2X3-DOPE in B16) and control group are indicated by asterisks (* *p* < 0.05 for P2000 and isRNA/P2000, ** *p* < 0.005 for isRNA/2X3-DOPE); ordinary two-way ANOVA, Dunnett’s multiple comparisons test.

P2000 PEG-containing and non-PEGylated 2X3-DOPE formulations were used for the investigation of the antiproliferative effects of isRNA/P2000 and isRNA/2X3-DOPE on tumor cells of different origins. Cell lines from different tissue origins, namely melanoma B16 and drug resistant lymphosarcoma RLS40, were selected for this experiment since they are tumorigenic in syngeneic mice and can be used further for antitumor activity assessment in vivo. The experimental scheme is depicted in Figure 4A. The cells were plated on 6-well plates one day before transfection in triplicate, and the number of living cells was assayed via WST-1 test at days 2–7.

During the observation period, RLS40 cells grew exponentially and did not show any statistically significant response to the treatment with isRNA/liposomes or liposomes alone. The number of living cells in isRNA/liposome-treated groups was equal to that in the vehicle group and, at some time points, even slightly exceeded the number of cells in the untreated control group (Figure 4B). A completely different picture is observed in the case of melanoma B16 cells: the number of living cells treated with isRNA/2X3-DOPE decreased profoundly in comparison with both the control group and the corresponding vehicle group the next day after transfection. P2000 slightly inhibited cell growth over the first three days after treatment, but in the following days, the cells recovered and grew similarly to other groups. The isRNA/P2000 complexes had almost no effect on the cell proliferation rate (Figure 4C). Such a difference in the behavior of “responding” and “non-responding” tumor cells can arise from both the characteristics of the cells themselves associated with their tissue origin, such as the presence of recognizing receptors and components of the downstream signaling pathways [31], as well as the different efficiency of isRNA accumulation in cells.

**Figure 5:**
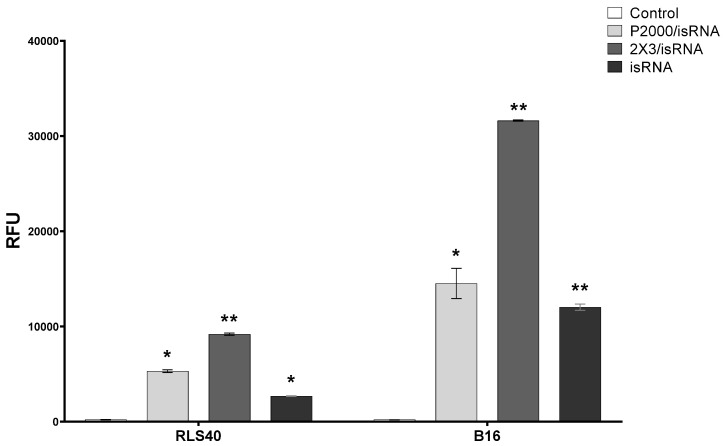
Accumulation of Cy5.5-isRNA and its complexes with liposomes in B16 and RLS40 cells. The accumulation was measured 4 h after transfection of Cy5.5-isRNA mediated by liposomes using flow cytometry. Statistically significant differences between experimental groups and control group are indicated by asterisks (* *p* < 0.005, ** *p* < 0.0001); ordinary two-way ANOVA, Dunnett’s multiple comparisons test. RFU—Relative Fluorescence Unite.

Cy5.5-labeled isRNA was used to compare the efficiency of its accumulation in B16 and RLS40 cells. The cells were transfected either with complexes or with isRNA alone, and after 4 h of incubation, the levels of isRNA accumulation in cells were measured using flow cytometry (Figure 5 and Figure S2). The results indicate that in all cases, the accumulation of free or complexed isRNA in B16 melanoma cells is at least twice as efficient as in RLS40 lymphosarcoma cells; nevertheless, the levels of accumulation of isRNA/2X3-DOPE complexes in RLS40 cells are quite significant. In both cell lines, isRNA accumulation mediated by 2X3-DOPE liposomes was much more efficient than that mediated by P2000 liposomes (Figure 5 and Figure S2). These data show that the patterns observed during isRNA delivery are similar to those for other types of TNA and are in good agreement with our recently obtained data showing that the incorporation of a PEG-lipoconjugate in liposomes reduces the efficiency of antisense oligonucleotides and plasmid DNA accumulation in cells in vitro [26].

**Figure 6:**
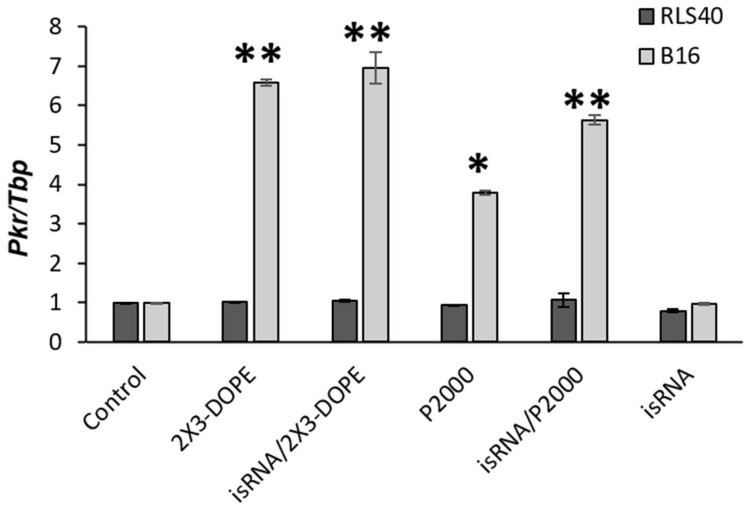
The levels of *Pkr* mRNA in RLS40 and B16 cells after treatment with isRNA/liposome or liposomes only. Relative expression of *Pkr* mRNA, normalized to the reference *Tbp* mRNA expression, was measured via RT-qPCR 24 h after transfection. *Pkr*/*Tbp* ratio in control was set as 1. Statistically significant differences between experimental groups and control group are indicated by asterisks (* *p* < 0.005, ** *p* < 0.0001); ordinary two-way ANOVA, Dunnett’s multiple comparisons test.

We evaluated the activation of a marker of immunostimulation, *Pkr* mRNA expression, to assess the immune response in cell lines of various origins. The B16 and RLS40 cells were transfected with isRNA/liposomes or liposomes only in comparison with control cells incubated with OptiMEM. The cells were incubated for 24 h; afterwards, mRNA was isolated from the cells, and the relative mRNA expression of *Pkr* was measured via RT-qPCR (Figure 6). *Pkr* mRNA was selected as a maker of immune response activation since this gene is overexpressed in the case of viral infections and encodes the dsRNA-dependent protein kinase R (PKR), an essential component of cellular antiviral defense in mammalian cells. Previously, we showed that isRNA activates the expression of this gene, and inhibition of PKR by 2-aminopurine blocks the antiproliferative activity of isRNA in human tumor cells [32].

The obtained data indicates that no pronounced activation of *Pkr* mRNA expression was observed in the treated RLS40 cells after isRNA transfection in comparison to the control cells. Conversely, melanoma B16 cells treated with isRNA/2X3-DOPE or 2X3-DOPE exhibited a significant 7-fold increase in *Pkr* mRNA expression levels. The complex isRNA/P2000 induced a 6-fold increase, while the effect of empty liposome P2000 produced a 4-fold increase in *Pkr* mRNA. isRNA without liposomes had no effect on marker expression. Thus, it can be concluded that RLS40 belongs to “non-responding” cell lines, which is due to the peculiarities of its tissue origin and the corresponding state of the transcriptome profile and not because the isRNA does not penetrate into these cells.

### 3.4. Antitumor Activity of isRNA/Liposomes in Mouse Models

**Figure 7:**
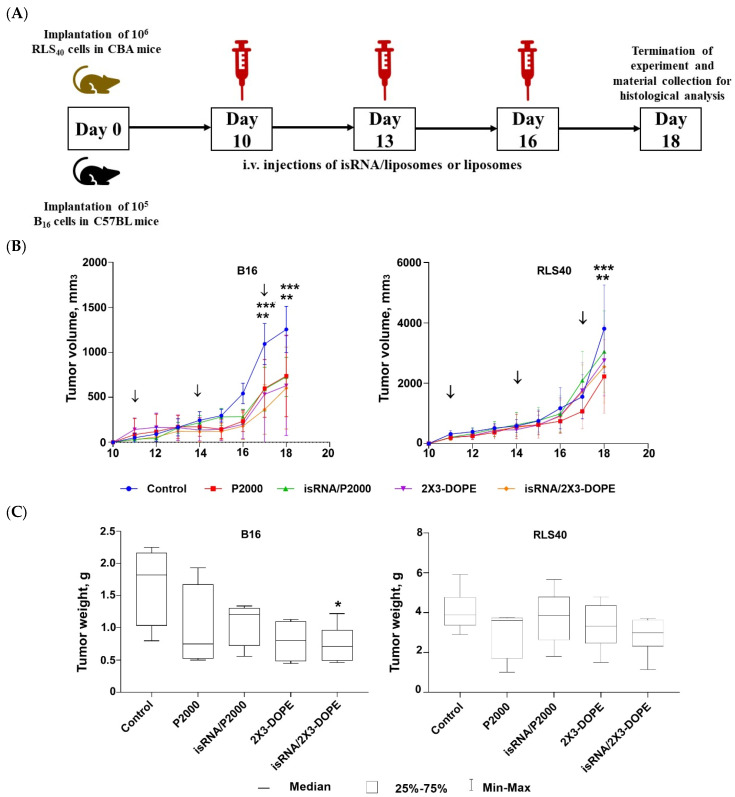
Antitumor activity of isRNA/liposomes in the lymphosarcoma RLS40 and melanoma B16 mouse models. (**A**) Experimental setup. (**B**) Dynamics of tumor growth (*n* = 5 mice per group). (**C**) Average tumor weight at the end point of the experiment. The arrows indicate the day when mice received injections. Statistically significant differences between experimental groups and the control group are indicated by asterisks (* *p* ˂ 0.05 compared to control group, ** *p* < 0.0005 for P2000 and isRNA/P2000 treated mice compared to the control group, *** *p* < 0.0001 for 2X3-DOPE and isRNA/2X3-DOPE compared to the control group); ordinary two-way ANOVA, Dunnett’s multiple comparison test.

In the transition from in vitro to in vivo, factors such as the biodistribution of the drug in the body and the rate of drug excretion from the bloodstream can affect or even determine the effectiveness of the antitumor action of isRNA. In addition, antitumor activity can be determined both from the effectiveness of the direct action of isRNA on tumor cells and from the effectiveness of isRNA-mediated activation of the immune system. In order to evaluate the antitumor effect of isRNA/liposomes in vivo, we used syngeneic mouse models relevant to studies of immunotherapies: melanoma B16 implanted in C57BL\6 mice and RLS40 in CBA mice. isRNA/liposomes or empty liposomes were administered intravenously at days 10, 13, and 16 after tumor implantation. Tumor growth was monitored, and at the end of the experiment, organs were collected for histological examination according to the experimental scheme presented in Figure 7A. The time intervals between injections were chosen in accordance with our earlier data on the duration of the interferon refractoriness period after isRNA administration [21].

Analysis of tumor growth dynamics demonstrated that the triple injections caused a delay in tumor growth and decreased its volume in the melanoma B16 model under the action of both isRNA/liposomes and the liposomes themselves. The most pronounced effect was observed after administration of isRNA/2X3-DOPE, which proved to have potent antitumor activity, causing a 2-fold decrease in the total tumor volume as compared to the control (Figure 7B, left graph). As for RLS40 lymphosarcoma-bearing mice, a slight but not considerable antitumor effect was observed (Figure 7B, right graph). A comparison of B16 tumor weights at the end of the experiment showed that only the tumors in mice that received isRNA/2X3-DOPE were statistically significantly smaller than those in the control group (Figure 7C, left graph). A downward trend in tumor volume was observed in the group treated with isRNA/P2000; however, the differences with the control are not statistically significant (Figure 7C, left graph). As expected in accordance with the growth kinetics of RLS tumors, their weight did not differ statistically significantly in all experimental groups (Figure 7C, right graph).

**Figure 8:**
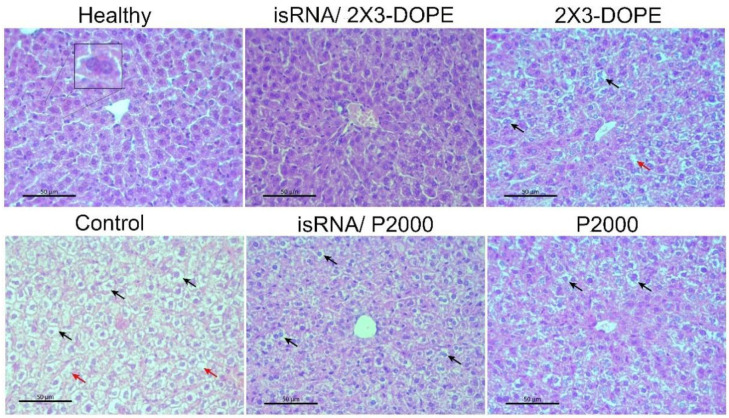
The effect of isRNA/liposome on the liver tissue of B16 melanoma-bearing mice. Representative histological images of liver tissues of healthy animals, control, non-treated mice, mice that received isRNA/2X3-DOPE or isRNA/P2000 complexes, and mice treated with 2X3-DOPE or P2000 in OptiMEM, respectively. Insets show binuclear hepatocytes (**left**), necrosis in the liver parenchyma (red arrows), and hepatocytes with dystrophy (black arrows). Hematoxylin and eosin staining, original magnification ×400. Scale bar corresponds to 50 µm.

Histological analyzes of the livers of healthy mice, as well as untreated, isRNA/liposomes or liposomes treated mice with B16 melanoma, were carried out to evaluate the possible toxicity of isRNA/liposomes treatment. The data demonstrate (Table 3, Figure 8) that B16 melanoma growth is accompanied by high liver toxicity manifesting in a pronounced rise in the total destructive changes in the liver parenchyma (3-fold increase) in the control non-treated group and the group that received 2X3-DOPE alone, representing the high levels of dystrophy and necrosis in comparison with the healthy control. Treatment with isRNA/2X3-DOPE and P2000 caused a 2-fold decrease in the total destructive changes in the liver parenchyma, as demonstrated by the reduction in both dystrophies and necrosis, while isRNA/P2000 caused a moderate decrease in the total destructive changes (Table 3). The data indicate that the decrease in destructive changes in the liver tissue is proportional to the decrease in tumor volume, and the administration to mice of isRNA/liposomes or empty liposomes has a hepatoprotective effect.

**Figure 9:**
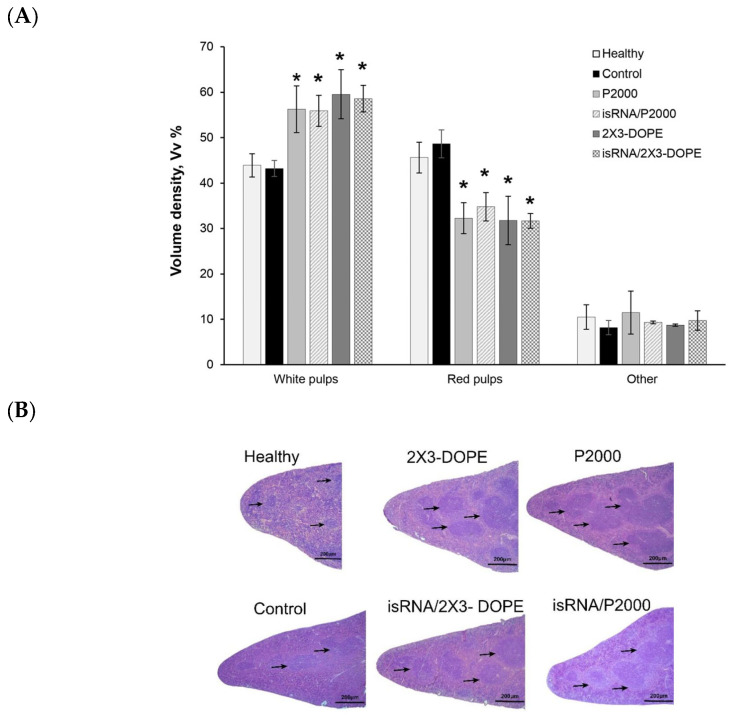
The effect of isRNA/liposome on spleen tissue from mice with melanoma B16. (**A**) Morphometry data of spleen from C57BL\6 mice bearing melanoma B16. Volume density of white and red pulp in spleen tissue after 3 injections of isRNA/liposomes or liposomes only. (**B**) Representative histological images of the spleen sections. Hematoxylin and eosin staining. Black arrows indicate lymphoid follicles in the spleen. Original magnification ×100. The data represent mean ± standard deviation (SD) calculated from measurements from five mice in each group. Statistically significant differences between experimental groups and the control and healthy groups are indicated by asterisks (* = *p* value < 0.001); ordinary two-way ANOVA, Dunnett’s multiple comparisons test. Scale bar corresponds to 200 µm.

The spleen is the most sensitive organ to immunostimulation; therefore, histological analysis was performed on spleen tissue. The spleen of control B16-bearing mice had a typical structure comparable to the spleen of healthy mice: the white pulp was moderately developed and consisted of lymphoid follicles (Figure 9). Three i.v. injections of isRNA/liposomes or liposomes induced a pronounced immune response in the spleen of treated mice, expressed as an increase in the volume density of the white pulp and the lymphoid follicles in the spleen (Figure 9A,B).

Although the CBA mice responded to isRNA/liposome administration by secreting high levels of type I interferons, this is not enough to significantly limit the growth of the RLS40 tumor, which itself does not respond to isRNA action. Comparison of the effectiveness of isRNA complexes with different liposomes on the development of B16 tumors in mice indicates that the effectiveness of the antiproliferative effect on tumor cells is of paramount importance for inhibiting tumor growth in syngeneic mice, which is accompanied by a decrease in liver toxicity and a pronounced immunostimulatory effect.

## 4. Discussion

Immunostimulatory nucleic acids acting as PRR agonists represent an attractive immunotherapeutic agent and have proven their efficacy as a monotherapy or an adjuvant therapy when combined with other therapies [33,34,35]. This approach has gained increasing interest over the last few decades, but the majority of nucleic acid molecules under study faced complications that hindered the advances in this field, such as their susceptibility to nuclease degradation in the bloodstream and tissues, their inability to penetrate into the cell with sufficient efficacy to interact with intracellular targets, and their rapid removal from the bloodstream due to their insufficient molecular weight [36,37]. In order to overcome these challenges, researchers developed different systems that were capable of delivering nucleic acids safely and effectively through these barriers to their molecular targets, protecting them from the degradative effect of nucleases and increasing their specificity. Synthetic, semi-synthetic, and natural materials have been developed that can encapsulate nucleic acids, such as liposomes, polymers, and various nanoparticles [38].

The current study used a specific 19-bp isRNA duplex with 3′-3-nt overhangs. This isRNA is one nucleotide longer than canonical siRNAs and has no substantial homology with mRNAs from mice or humans; hence, it cannot change the gene expression pattern via an RNA interference mechanism. The immunostimulatory, interferon-inducing, antiproliferative, antitumor, and antiviral activities of isRNA were examined in vitro and in vivo [21,26]. We have shown earlier that the immunostimulatory activity of this isRNA is manifested only when delivery systems are used, which is consistent with the involvement of intracellular molecular targets of RIG-I and PKR in the antiproliferative action of isRNA [39]. Liposomes represent a very promising delivery system because of their cell membrane-like structure, which significantly enhances cellular uptake and accumulation and allows for efficient cell affinity [40,41,42,43]. In this study, for the first time, a detailed profiling of cytokines secreted in mouse blood under the action of complexes of liposomes with isRNA was carried out, and it was shown that the main cytokines, the levels of which were significantly increased, are the cytokine monocyte chemoattractant protein 1 (MCP-1) and interferons α and γ; moderate activation of interferon β, pro-inflammatory cytokines IL-6, and to a lower degree, IL-1α and TNF-α are also observed (Figure 3). Our data on cytokine profiling demonstrate surprisingly high levels of MCP-1, also known as the chemokine (C-C motif) ligand 2 (CCL2). Relations between MCP-1 and both type I and type II interferons in inflammation and antiviral defenses were studied in the work of Hokeness et al., 2005 [44] on a mouse cytomegalovirus (CMV) infection model. They analyzed the MCP-1 levels in mice that were either immunocompetent or deficient in IFN-α/β functions (IFN-α/βR^−^), and it was shown that the responses were decreased 5-fold in IFN-α/βR^−^ mice in comparison to immunocompetent animals. Gill et al., 2006 [45] discussed in their work how multiple waves of IFNs can affect MCP-1 levels in the course of herpes simplex virus (HSV-2) infection. The early wave of IFN-β (12 h after infection) led to the induction of MCP-1-mediated inflammatory monocyte recruitment, subsequently leading to IL-18-induced NK cell IFN-γ production. The second wave of type I IFNs was accompanied by a significant rise in IFN-γ from NK cells at 48 h post-infection, which was negatively regulated by type I IFN, and our data correlate to these findings as we have shown an increase in the level of both type I and II interferons.

The modular structure of such delivery systems makes it possible to control not only the size and charge of their complexes with nucleic acids but also to change their properties using surface decoration by adding miscellaneous lipoconjugates [46]. The main functions of lipoconjugates in liposomes are aimed at providing targeted delivery to certain types of cells expressing the corresponding receptors, protecting liposomes and their complexes from absorption by RES cells, aggregation, and opsonization, as well as controlling their size and surface charge.

Targeted delivery methods include the use of antibodies, aptamers, and ligands of receptors that are overexpressed on the surface of target cells; folate is one of the most widely utilized and studied ligands [25,29]. As we have previously shown, when folate lipoconjugate is present in 2X3-DOPE core liposomes, siRNA accumulation in tumor cells that are overexpressing folate receptors increases [29]. In the current investigation, we delivered isRNA using folate-containing liposomes and discovered that the folate lipoconjugate decreased the efficacy of interferon induction (Figure 2), showing that interferon-producing cells do not have an elevated level of folate receptors. It is not yet possible to choose particular ligands for interferon-producing cells, as they have not been discovered for isRNA at this point in the investigation.

In order to increase the circulation time and stability of the drug in the blood stream, the surface of the liposomes was modified by adding a protective layer consisting of PEG [47]. A comparison of the effectiveness of the interferon-inducing action of isRNA delivered in mice and primary cultures of human mononuclear cells in complexes with liposomes containing PEG-lipoconjugates of various lengths and structures showed that the choice of the most optimal compositions depends to a certain extent on the experimental object. In general, it is preferable to use lipoconjugates with longer PEG, whereas increasing the density of the shield by increasing the content of the lipoconjugate or folding it into a loop-like structure does not provide a visible advantage (Figure 2, Figure 3 and Figure 4). It should be noted that liposomes containing long PEG demonstrate a significant advantage both in the efficiency of interferon induction in the presence of isRNA and in the specificity of action in the absence of isRNA (Figure 4).

Kabilova et al. reported in 2018 that an increase in the length of PEG in liposomes reduces their transfection efficiency in vitro but increases the time of circulation in the bloodstream in vivo [26]. The latter feature may be especially important for its effect on interferon-producing cells in the body. This is consistent with our findings, which showed that liposomes P2000 induce IFN-α in mice significantly more efficiently than P1500 and P800 (Figure 2 and Figure 3). While transfecting tumor cells in vitro non-PEGylated liposomes (2X3-DOPE) accumulated in cells faster and better than PEGylated liposomes (P2000).

Cytokine profiling revealed that the spectrum of cytokines activated by isRNA in complex with liposomes also depends on the liposome’s composition (Figure 3). Thus, the greatest activation of MCP-1, INF-α, β, and γ occurs under the action of isRNA complexes with P1500 and P2000 liposomes, while isRNA complexes with P800 caused significant activation of MCP-1 and INF-γ but not INF-α or β. Since type I interferons are important antiviral and antitumor agents, the ability to activate them is important for the delivery system.

An increasing body of literature reports the usage of different immunostimulatory nucleic acids in immunotherapy and explains how using different delivery systems results in different responses [16,40,48,49]. In the present study, usage of isRNA complexed with liposomes P800 and P1500 induced a noticeable rise in IFN-γ levels in comparison to the control group (around 750 pg/mL). This indicates the participation of different molecular sensors of isRNA, depending on the intracellular localization of the cargo delivered by the liposomal system; it is not possible to exclude the participation of endosomal sensors, such as TLR7/8 [50]. Due to their potent innate immunostimulatory properties, TLR7/8 agonists have shown impressive results as anti-cancer therapy when administered systematically [51]. Since these receptors are endosomal, their agonists need to be internalized into the cells in order to activate them. In the work of Obermann et al., cholesterol-conjugated RNA (double-stranded version of immunostimulatory RNA40-chol) formed nanoparticles which were compared to RNA-liposome complexes and found to be more efficient in inducing type I interferons from human and murine plasmacytoid dendritic cells as well as proinflammatory cytokine production (e.g., TNF-α, IL12p70, or IL-6) in human monocytes in vitro [52].

An important target for interferon-inducing therapeutics is the retinoic acid-inducible gene-I (RIG-I) receptor, which detects the presence of dsRNA with a 5′ triphosphate (3pRNA) in the cytosol and initiates an immune response to eliminate threats [16]. Synthetic dsRNA molecules complexed with different delivery systems have been studied as potential therapeutics. In a study conducted by M. Jacobson et al., the activation of the RIG-I receptor by 5′-3pRNA delivered by polymeric nanoparticles (PEG-block-(DMAEMA-co-AnMA) polymers, where AnMA is an alkyl methacrylate monomer ranging from *n* = 2–12 carbons of variable composition) intravenously was achieved [53]. The researchers in this study identified four lead carriers (4–50, 6–40, 8–40, and 10–40, where the first number refers to the alkyl chain length and the second number refers to the percentage of hydrophobic monomer), which considerably improved the immunostimulatory activity of 5′-triphospate RNA RIG-I ligands in vitro and in vivo. It caused activation and exhibited a notable rise in the levels of IFN-α in serum and elevated expression of *Ifnb1* and *Cxcl10* in major clearance organs.

PKR is another important sensor that recognizes isRNA [54]. We have previously shown that the use of the PKR protein inhibitor 2-aminopurine or hairpin RNA targeting the *PKR* gene prevents the antiproliferative effects of isRNA on tumor cells when delivered with 2X3-DOPE [39]. In previous work, anti-tumor and anti-proliferative activity of isRNA delivered by 2X3-DOPE liposomes or Lipofectamine 2000 was proven against melanoma and hepatoma, caused a decrease in tumor size in comparison to the control or mock-treated group, and exhibited anti-metastatic activity, and the observed effect correlates with transfection efficiency [21]. In another work, it was shown that the use of PEGylated liposomes to deliver isRNA provides an advantage for antiviral activity and increases the effectiveness of inhibiting the development of influenza infection in mice, and the observed effect correlates with the efficiency of interferon induction [55].

We compared the antiproliferative and antitumor effects of delivery systems with opposite effects (2X3-DOPE and P2000) in B16 melanoma and RLS lymphosarcoma models to determine whether the ability to activate interferon production or transfection efficiency is more important for this effect. We found that isRNA/2X3-DOPE complexes have a pronounced antiproliferative effect on B16 in vitro as well as an antitumor and hepatoprotective effect in vivo (Figure 7 and Figure 8, Table 3), while isRNA/P2000 caused a moderate decrease in tumor size in comparison to the untreated mice and also had a noteworthy antiproliferative effect in vitro. The results obtained showed that the direct effect on tumor cells has a more significant effect on tumor growth than the induction of interferon and activation of the immune system. Thus, it can be concluded that the main mechanism of the antitumor effect of isRNA/liposome complexes on B16 tumors is a decrease in tumor growth due to the direct antiproliferative effect of isRNA on tumor cells. This effect is associated with a slowdown in the rate of cell growth or even its complete stop under the action of isRNA, due to the activation of the innate immune system. We have previously demonstrated that the selective silencing of the PKR and RIG-I genes in human KB-3-1 and A549 cells prevented the antiproliferative effect of isRNA, although the expression of MDA5 and IRF3 is not critical for this effect [39].

We found that one of the tumor models we selected, lymphosarcoma RLS40, did not respond to isRNA complexes with the studied liposomes (Figure 7), and it did not activate expression of the immune response marker *Pkr* (Figure 6). This situation reflects the situation that exists in clinics, where there are “responders” and “non-responders” to certain types of treatment of the tumor [56]. The reason for this in this case was not determined in this study; it is probably associated with both the tissue origin of tumor cells and the less effective accumulation of isRNA complexes in them (Figure 5). The delivery system did not exhibit toxic effects and proved its ability to activate innate immunity; therefore, it is possible to consider it a potential drug for treating melanoma.

Thus, we demonstrated that the structure of the delivery system plays a vital role in determining the immune response to isRNA; adding lipoconjugates can change the spectrum and the levels of the effects produced by immunostimulating RNA and allows for the choice of a delivery system depending on the desired effect.

## Figures and Tables

**Table 1:** Primers used for RT-qPCR.

Gene	Primer/Probe	Sequence 5′–3′
*Pkr*	probe	((5,6)-FAM)-ATATAACACGGAGAAGGCGGAGCAC-BHQ1
	forward	TGGCTTAGGTGGATTTGGTC
	reverse	GTTGACGTGATTGAGTTCTGC
*Tbp*	probe	5′-((5,6)-FAM)-CACTCCTGCCACACCAGCTTCT–BHQ1
	Forward	CACCAATGACTCCTATGACCC
	reverse	CAAGTTTACAGCCAAGATTCACG

**Table 2:** Hydrodynamic diameters, polydispersity index, and ζ-potentials of lipoplexes formed by isRNA and cationic liposomes.

Liposome	Composition, mol%	N/P ^1^	Size, d nm	Z, mV	PdI
2X3-DOPE	2X3/DOPE (50/50)	-	125.6 ± 0.6	42.5 ± 8.4	0.2
		6/1	218.8 ± 4.6	24.0 ± 0.3	0.4
P800	2X3/DOPE/P800 (49/49/2)	-	99.8 ± 3.1	40.8 ± 2.9	0.4
		6/1	156.0 ± 28.1	33.9 ± 0.6	0.4
P800 (4%)	2X3/DOPE/P800 (48/48/4)	-	61.5 ± 2.1	40.7 ± 1.2	0.4
		6/1	125.4 ± 3.4	22.1 ± 1.8	0.3
P800 + F12 (2%)	2X3/DOPE/P800/F12 (48/48/2/2)	-	57.9 ± 1.0	40.6 ± 2.4	0.3
		6/1	172.4 ± 13.4	40.2 ± 0.3	0.4
diP800	2X3/DOPE/diP800 (49/49/2)	-	99.8 ± 3.1	40.8 ± 2.9	0.5
		6/1	235.3 ± 31.9	38.4 ± 0.9	0.3
diP800 (4%)	2X3/DOPE/diP800 (48/48/4)	-	127.3 ± 1.5	48.9 ± 0.9	0.5
		6/1	111.8 ± 2.5	23.2 ± 4.1	0.4
diP800 + F12 (2%)	2X3/DOPE/diP800/F12 (48/48/2/2)	-	110.6 ± 2.5	47.7 ± 0.4	0.3
		6/1	218.8 ± 9.4	36.2 ± 1.1	0.3
P1500	2X3/DOPE/P1500 (49/49/2)	-	102.0 ± 6.8	21.4 ± 2.1	0.3
		6/1	223.1 ± 7.7	25.3 ± 1.9	0.4
diP1500	2X3/DOPE/diP1500 (49/49/2)	-	98.0 ± 4.3	51.3 ± 1.1	0.4
		6/1	119.2 ± 1.5	10.3 ± 1.1	0.4
P2000	2X3/DOPE/P2000 (49/49/2)	-	67.4 ± 2.1	47.3 ± 1.3	0.4
		6/1	114.3 ± 2.3	13.3 ± 2.1	0.6
diP2000	2X3/DOPE/diP2000 (49/49/2)	-	109.8 ± 1.2	54.1 ± 2.7	0.3
		6/1	129.6 ± 11.4	15.1 ± 1.1	0.4
F12	2X3/DOPE/F12 (49/49/2)	-	77.3 ± 9.5	35.7 ± 5.6	0.4
		6/1	170.8 ± 4.8	34.5 ± 0.4	0.4

^1^ Nitrogen to phosphorous ratio in isRNA/liposome complex.

**Table 3:** Morphological changes ^1^ to the liver tissue of healthy mice and mice with B16 melanoma without treatment (Control) and after isRNA/liposome or liposome administration.

	Healthy	Control	2X3-DOPE	isRNA/2X3-DOPE	P2000	isRNA/P2000
Normal hepatocytes, Vv, %	80.0 ± 1.7 ***	54.9± 16.5 ###	52.7 ± 9.9 ###	71.8 ± 6.3 **	69.3 ± 16.6 *	63.4 ± 2.2 ##
Dystrophy, Vv, %	6.3 ± 0.7 ***	32.3 ± 14.9 ###	30.9± 11.6 ###	15.3 ± 4.9 ** #	17.2 ± 16.4 * ##	21.3 ± 1.9 ###
**Necrosis, Vv, %**	**2.0 ± 0.7**	**2.1 ± 1.7**	**4.9 ± 1.4**	**2.1 ± 1.7**	**0.7 ± 0.1**	**2.2 ± 0.6**
Total destructive changes, Vv, %	9.4 ± 1.2	34.4 ± 16.6	35.7 ± 13.0	17.1 + 6.6	17.7 ± 16.5	23.5 ± 2.6
Other, Vv, %	11.7 ± 1.3	10.5 ± 0.9	11.6 ± 1.1	10.7 ± 0.4	12.9 ± 1.1	13.1 ± 0.3
Binuclear hepatocytes, Nv	3.5 ± 1.2	14.2 ±6.2	19.4 ± 5.5	14.7 ± 4.9	20.4 ± 7.7	9.0 ± 0.4

^1^ Average mean ± SD is presented in the table; statistically significant differences between experimental groups and the control group are indicated by asterisks and between experimental groups and the healthy group are indicated by hash (* or # *p* ˂ 0.05, ** or **##** *p* < 0.01, *** or **###** *p* < 0.0001); ordinary two-way ANOVA, Dunnett’s multiple comparison test.

## Data Availability

The data presented in this study are available in this article (and Supplementary Materials).

## Supplementary Materials

The following supporting information can be downloaded at: https://www.mdpi.com/article/10.3390/pharmaceutics15092184/s1. Table S1: Cytokine profiling measured using LEGEND plex™ Mouse Inflammation Panel (13-plex) kit after injections with isRNA/liposomes of different structures. The data represent mean ± standard deviation (SD) calculated from measurements from at least three mice. Statistically significant differences between experimental groups and the control group are indicated by asterisks (*** = *p* value ≤ 0.0001, ** = *p* value < 0.001, * = *p* value < 0.05); ordinary Two-way ANOVA, Dunnett’s multiple comparisons test. Figure S1: Cytokine levels in human PBMC primary cell culture after transfection with isRNA/liposomes. The levels of IFN-α (A) and TNF-α (B) 6 and 24 h after transfection, the levels of IL-6 (C) 16 and 24 h after transfection were measured via ELISA. The data represent mean ± standard deviation (SD). Statistically significant differences between samples treated with isRNA/liposomes and the samples treated with only liposomes are indicated by asterisk (* = *p* value < 0.05, ** = *p* value < 0.01, *** = *p* value < 0.001); ordinary two-way ANOVA, Dunnett’s multiple comparisons test. Figure S2: Representative flow histograms of Cy5.5-isRNA and its complexes with liposome accumulation in B16 and RLS40 cells. The accumulation was measured 4 h after transfection with Cy5.5-isRNA mediated by liposomes. Red—untreated cells, —cells incubated with -siRNA, Yellow—cells incubated with Cy5.5-isRNA/P2000 complexes, Blue—cells incubated with Cy5.5-isRNA/2X3-DOPE complexes.

## References

[1] Debela D.T., Muzazu S.G., Heraro K.D., Ndalama M.T., Mesele B.W., Haile D.C., Kitui S.K., Manyazewal T. (2021). New Approaches and Procedures for Cancer Treatment: Current Perspectives. SAGE Open Med..

[2] Side Effects of Cancer Treatment—NCI. https://www.cancer.gov/about-cancer/treatment/side-effects.

[3] Robinson J.K., Fisher S.G. (2000). Recurrent Basal Cell Carcinoma After Incomplete Resection. Arch. Dermatol..

[4] Naran K., Nundalall T., Chetty S., Barth S. (2018). Principles of Immunotherapy: Implications for Treatment Strategies in Cancer and Infectious Diseases. Front. Microbiol..

[5] Zhang Y., Zhang Z. (2020). The History and Advances in Cancer Immunotherapy: Understanding the Characteristics of Tumor-Infiltrating Immune Cells and Their Therapeutic Implications. Cell Mol. Immunol..

[6] Petitprez F., Meylan M., de Reyniès A., Sautès-Fridman C., Fridman W.H. (2020). The Tumor Microenvironment in the Response to Immune Checkpoint Blockade Therapies. Front. Immunol..

[7] Titov A., Zmievskaya E., Ganeeva I., Valiullina A., Petukhov A., Rakhmatullina A., Miftakhova R., Fainshtein M., Rizvanov A., Bulatov E. (2021). Adoptive Immunotherapy beyond CAR T-Cells. Cancers.

[8] Shen T., Zhang Y., Zhou S., Lin S., Zhang X.-B., Zhu G. (2020). Nucleic Acid Immunotherapeutics for Cancer. ACS Appl. Bio Mater..

[9] Sridharan K., Gogtay N.J. (2016). Therapeutic Nucleic Acids: Current Clinical Status. Br. J. Clin. Pharmacol..

[10] Yamada Y. (2021). Nucleic Acid Drugs—Current Status, Issues, and Expectations for Exosomes. Cancers.

[11] Herwald H., Egesten A. (2016). On PAMPs and DAMPs. J. Innate Immun..

[12] Li N., Geng C., Hou S., Fan H., Gong Y. (2020). Damage-Associated Molecular Patterns and Their Signaling Pathways in Primary Blast Lung Injury: New Research Progress and Future Directions. Int. J. Mol. Sci..

[13] Amarante-Mendes G.P., Adjemian S., Branco L.M., Zanetti L.C., Weinlich R., Bortoluci K.R. (2018). Pattern Recognition Receptors and the Host Cell Death Molecular Machinery. Front. Immunol..

[14] He X., Jia H., Jing Z., Liu D. (2013). Recognition of Pathogen-Associated Nucleic Acids by Endosomal Nucleic Acid-Sensing Toll-like Receptors. Acta Biochim. Biophys. Sin..

[15] Okude H., Ori D., Kawai T. (2021). Signaling Through Nucleic Acid Sensors and Their Roles in Inflammatory Diseases. Front. Immunol..

[16] Bishani A., Chernolovskaya E.L. (2021). Activation of Innate Immunity by Therapeutic Nucleic Acids. Int. J. Mol. Sci..

[17] Hasan U.A., Caux C., Perrot I., Doffin A.-C., Menetrier-Caux C., Trinchieri G., Tommasino M., Vlach J. (2007). Cell Proliferation and Survival Induced by Toll-like Receptors Is Antagonized by Type I IFNs. Proc. Natl. Acad. Sci. USA.

[18] Kolte A., Baradia D., Patil S., Vhora I., Kore G., Misra A. (2014). PEG—A Versatile Conjugating Ligand for Drugs and Drug Delivery Systems. J. Control. Release Off. J. Control. Release Soc..

[19] Samaridou E., Heyes J., Lutwyche P. (2020). Lipid Nanoparticles for Nucleic Acid Delivery: Current Perspectives. Adv. Drug Deliv. Rev..

[20] Tran P., Weldemichael T., Liu Z., Li H. (2022). Delivery of Oligonucleotides: Efficiency with Lipid Conjugation and Clinical Outcome. Pharmaceutics.

[21] Kabilova T.O., Sen’kova A.V., Nikolin V.P., Popova N.A., Zenkova M.A., Vlassov V.V., Chernolovskaya E.L. (2016). Antitumor and Antimetastatic Effect of Small Immunostimulatory RNA against B16 Melanoma in Mice. PLoS ONE.

[22] Maslov M.A., Kabilova T.O., Petukhov I.A., Morozova N.G., Serebrennikova G.A., Vlassov V.V., Zenkova M.A. (2012). Novel Cholesterol Spermine Conjugates Provide Efficient Cellular Delivery of Plasmid DNA and Small Interfering RNA. J. Control. Release.

[23] Vysochinskaya V., Shishlyannikov S., Zabrodskaya Y., Shmendel E., Klotchenko S., Dobrovolskaya O., Gavrilova N., Makarova D., Plotnikova M., Elpaeva E. (2023). Influence of Lipid Composition of Cationic Liposomes 2X3-DOPE on MRNA Delivery into Eukaryotic Cells. Pharmaceutics.

[24] Shmendel E.V., Bakhareva S.A., Makarova D.M., Chernikov I.V., Morozova N.G., Chernolovskaya E.L., Zenkova M.A., Maslov M.A. (2020). Uncharged Gemini-Amphiphiles as Components of Cationic Liposomes for Delivery of Nucleic Acids. Russ. J. Bioorganic Chem..

[25] Gladkikh D.V., Sen Kova A.V., Chernikov I.V., Kabilova T.O., Popova N.A., Nikolin V.P., Shmendel E.V., Maslov M.A., Vlassov V.V., Zenkova M.A. (2021). Folate-Equipped Cationic Liposomes Deliver Anti-MDR1-SiRNA to the Tumor and Increase the Efficiency of Chemotherapy. Pharmaceutics.

[26] Kabilova T., Shmendel E., Gladkikh D., Morozova N., Maslov M., Chernolovskaya E., Vlassov V., Zenkova M. (2018). Novel PEGylated Liposomes Enhance Immunostimulating Activity of IsRNA. Molecules.

[27] Luneva A.S., Puchkov P.A., Shmendel E.V., Zenkova M.A., Kuzevanova A.Y., Alimov A.A., Karpukhin A.V., Maslov M.A. (2018). Optimization of the Technology for the Preparation of Cationic Liposomes for the Delivery of Nucleic Acids. Russ. J. Bioorganic Chem..

[28] Petukhov I.A., Maslov M.A., Morozova N.G., Serebrennikova G.A. (2010). Synthesis of Polycationic Lipids Based on Cholesterol and Spermine. Russ. Chem. Bull..

[29] Kabilova T.O., Shmendel E.V., Gladkikh D.V., Chernolovskaya E.L., Markov O.V., Morozova N.G., Maslov M.A., Zenkova M.A. (2018). Targeted Delivery of Nucleic Acids into Xenograft Tumors Mediated by Novel Folate-Equipped Liposomes. Eur. J. Pharm. Biopharm..

[30] Complete Mixed Feeds for Laboratory Animals. Specification. https://docs.cntd.ru/document/1200167514.

[31] Housman G., Byler S., Heerboth S., Lapinska K., Longacre M., Snyder N., Sarkar S. (2014). Drug Resistance in Cancer: An Overview. Cancers.

[32] Kabilova T.O., Vladimirova A.V., Zenkova M.A., Chernolovskaya E.L., Vlassov V.V. (2011). Antiproliferative and Interferon-Inducing Activities of Unique Short Double-Stranded RNA. Dokl. Biochem. Biophys..

[33] Ong G.H., Lian B.S.X., Kawasaki T., Kawai T. (2021). Exploration of Pattern Recognition Receptor Agonists as Candidate Adjuvants. Front. Cell Infect. Microbiol..

[34] Yang J.-X., Tseng J.-C., Yu G.-Y., Luo Y., Huang C.-Y.F., Hong Y.-R., Chuang T.-H. (2022). Recent Advances in the Development of Toll-like Receptor Agonist-Based Vaccine Adjuvants for Infectious Diseases. Pharmaceutics.

[35] Burn O.K., Prasit K.K., Hermans I.F. (2020). Modulating the Tumour Microenvironment by Intratumoural Injection of Pattern Recognition Receptor Agonists. Cancers.

[36] Elsabahy M., Nazarali A., Foldvari M. (2011). Non-Viral Nucleic Acid Delivery: Key Challenges and Future Directions. Curr. Drug Deliv..

[37] Ingle R.G., Fang W.-J. (2023). An Overview of the Stability and Delivery Challenges of Commercial Nucleic Acid Therapeutics. Pharmaceutics.

[38] Joseph T.M., Kar Mahapatra D., Esmaeili A., Piszczyk Ł., Hasanin M.S., Kattali M., Haponiuk J., Thomas S. (2023). Nanoparticles: Taking a Unique Position in Medicine. Nanomaterials.

[39] Zharkov M.I., Zenkova M.A., Vlassov V.V., Chernolovskaya E.L. (2019). Molecular Mechanism of the Antiproliferative Activity of Short Immunostimulating DsRNA. Front. Oncol..

[40] Yan Y., Liu X.-Y., Lu A., Wang X.-Y., Jiang L.-X., Wang J.-C. (2022). Non-Viral Vectors for RNA Delivery. J. Control. Release.

[41] Tenchov R., Bird R., Curtze A.E., Zhou Q. (2021). Lipid Nanoparticles—From Liposomes to MRNA Vaccine Delivery, a Landscape of Research Diversity and Advancement. ACS Nano.

[42] Hald Albertsen C., Kulkarni J.A., Witzigmann D., Lind M., Petersson K., Simonsen J.B. (2022). The Role of Lipid Components in Lipid Nanoparticles for Vaccines and Gene Therapy. Adv. Drug Deliv. Rev..

[43] Álvarez-Benedicto E., Farbiak L., Márquez Ramírez M., Wang X., Johnson L.T., Mian O., Guerrero E.D., Siegwart D.J. (2022). Optimization of Phospholipid Chemistry for Improved Lipid Nanoparticle (LNP) Delivery of Messenger RNA (MRNA). Biomater. Sci..

[44] Hokeness K.L., Kuziel W.A., Biron C.A., Salazar-Mather T.P. (2005). Monocyte Chemoattractant Protein-1 and CCR2 Interactions Are Required for IFN-Alpha/Beta-Induced Inflammatory Responses and Antiviral Defense in Liver. J. Immunol..

[45] Gill N., Deacon P.M., Lichty B., Mossman K.L., Ashkar A.A. (2006). Induction of Innate Immunity against Herpes Simplex Virus Type 2 Infection via Local Delivery of Toll-Like Receptor Ligands Correlates with Beta Interferon Production. J. Virol..

[46] Torres-Vanegas J.D., Cruz J.C., Reyes L.H. (2021). Delivery Systems for Nucleic Acids and Proteins: Barriers, Cell Capture Pathways and Nanocarriers. Pharmaceutics.

[47] Nunes S.S., Fernandes R.S., Cavalcante C.H., da Costa César I., Leite E.A., Lopes S.C.A., Ferretti A., Rubello D., Townsend D.M., de Oliveira M.C. (2019). Influence of PEG Coating on the Biodistribution and Tumor Accumulation of PH-Sensitive Liposomes. Drug Deliv. Transl. Res..

[48] Meng F., Wang J., Yeo Y. (2022). Nucleic Acid and Oligonucleotide Delivery for Activating Innate Immunity in Cancer Immunotherapy. J. Control. Release.

[49] Mendes B.B., Conniot J., Avital A., Yao D., Jiang X., Zhou X., Sharf-Pauker N., Xiao Y., Adir O., Liang H. (2022). Nanodelivery of Nucleic Acids. Nat. Rev. Methods Primers.

[50] Kiaie S.H., Majidi Zolbanin N., Ahmadi A., Bagherifar R., Valizadeh H., Kashanchi F., Jafari R. (2022). Recent Advances in MRNA-LNP Therapeutics: Immunological and Pharmacological Aspects. J. Nanobiotechnol..

[51] Stickdorn J., Stein L., Arnold-Schild D., Hahlbrock J., Medina-Montano C., Bartneck J., Ziß T., Montermann E., Kappel C., Hobernik D. (2022). Systemically Administered TLR7/8 Agonist and Antigen-Conjugated Nanogels Govern Immune Responses against Tumors. ACS Nano.

[52] Obermann H.-L., Lederbogen I.I., Steele J., Dorna J., Sander L.E., Engelhardt K., Bakowsky U., Kaufmann A., Bauer S. (2022). RNA-Cholesterol Nanoparticles Function as Potent Immune Activators via TLR7 and TLR8. Front. Immunol..

[53] Jacobson M.E., Becker K.W., Palmer C.R., Pastora L.E., Fletcher R.B., Collins K.A., Fedorova O., Duvall C.L., Pyle A.M., Wilson J.T. (2020). Structural Optimization of Polymeric Carriers to Enhance the Immunostimulatory Activity of Molecularly Defined RIG-I Agonists. ACS Cent. Sci..

[54] Kim Y., Park J., Kim S., Kim M., Kang M.-G., Kwak C., Kang M., Kim B., Rhee H.-W., Kim V.N. (2018). PKR Senses Nuclear and Mitochondrial Signals by Interacting with Endogenous Double-Stranded RNAs. Mol. Cell.

[55] Goncharova E.P., Sen’kova A.V., Savin I.A., Kabilova T.O., Zenkova M.A., Vlassov V.V., Chernolovskaya E.L. (2020). Immunostimulating RNA Delivered by P1500 PEGylated Cationic Liposomes Limits Influenza Infection in C57Bl/6 Mice. Pharmaceutics.

[56] Dalgleish A.G. (2019). Why Do the Majority of Patients Not Respond at All, or Only Partially or Transiently, to Immunotherapy?. Expert Rev. Anticancer Ther..

