# Supplementary material for: Influence of the Composition of Cationic Liposomes on the Performance of Cargo Immunostimulatory RNA
Source: Pharmaceutics. 2023 Aug 23;15(9):2184. doi: 10.3390/pharmaceutics15092184 (PMC10535620; doi:10.3390/pharmaceutics15092184)
Supplement: Supplementary file 1 [file pharmaceutics-15-02184-s001.zip › pharmaceutics-2574814-supplementary.pdf]

Table S1: Cytokines profiling measured using LEGEND plex™ Mouse Inflammation Panel (13-plex) kit after injections with isRNA + liposomes of different structures. The data represent mean ± standard deviation (SD) calculated from measurements from at least three mice. Statistically significant differences between experimental groups and the Control group are indicated by asterisk (*** = P value ≤0.0001, ** = P value <0.001, * = P value <0.05); ordinary Two-way ANOVA, Dunnett's multiple comparisons test.

	CTRL	2X3	F12	P800	P800(4%)	P800+F12	diP800	diP800(4%)	diP800+F12	P1500	diP1500	P2000	diP2000
**IL-23**	0	29.11±10.15	0	0	0	0	0	4.85±11.88 ***	13.39±20.78	0	0	0	38.2±15.39
**IL-1α**	58.33±15.75	151.44±14.92	31.26±2.15	90.87±7.94	47.05±21.28	33.38±1.36	254.06±70.08	331.306±314.91	58.85±2.80	74.35±15.89	58.31±33.98	25.85±10.98	59.04±20.56
**IFN-γ**	0	180.18±63.75 *	451.09±138.24 ***	832.43±340.51 ***	608.68±67.84 ***	279.41±10.82 **	286.02±76.15 ***	567.56±31.56 ***	273.59±59.38 ***	753.63±71.64 ***	105.73±26.17	191.45±80.02 *	0
**TNF-α**	0	124.31±25.19	79.48±34.03	140.36±49.10	150.53±32.78	0	285.8±14.31	160.67±82.00 **	66.09±19.29 ***	188.05±116.97	0	79.75±0	106.45+56.60 ***
**MCP-1**	0	483.68±146.37 ***	887.88±243.91***	1488.43±131.06***	919.51±183.54 ***	70.41±5.51	903.56±169.44 ***	1220.31±241.35 ***	672.23±62.69 ***	2101.24±857.31 ***	773.48±214.73 ***	1239.09±370.26 ***	586.81±120.2
**IL-12p70**	0	15.04±6.47	19.95±6.46	15.26±3.45	29.69±5.47	0	9.85±1.55	26.41±5.59	13.85±10.70	37.57±16.18	0	167.8±0	0
**IL-1β**	0	0	0	0	0	0	110.28±56.08	0	23.68±12.00	0	0	0	0
**IL-10**	0	103.1±10.61	0	0	0	0	248.29±147.71 **	0	52.47±42.10	0	0	196.27±25.44 **	0
**IL-6**	3.98±1.46	120.31±56.47	85.11±41.76	181.53±48.86 *	100.72±51.04	87.77±5.05	228.63±20.08	127.65±38.89	103.11±6.48	277.3±64.89 ***	180.88±100.71 *	193.58±82.48	171.87±65.99
**IL-27**	0	236.5±61.99	0	22.38±0	0	0	95.44±17.82	0	65.77±55.68	139.66±107.37 *	0	0	0
**IL-17A**	0	3.05±1.20	0.57±0	0	0	0	14.81±9.20	0	4.17±3.89	0	0	0	0
**IFN-β**	0	28.26±13.56	97.97±36.84	118.44±21.99	142.57±24.90	0	31.88±9.52	140.85±20.75	46.13±34.58	330.35±55.69 ***	122.97±32.03	274.98±38.28	49.62±30.55
**GM-CSF**	3.31±5.17	7.45+1.71	0	0	0	0	23.69±1.56	0	0	0	0	63.79±0.1	7.32±1.55

A

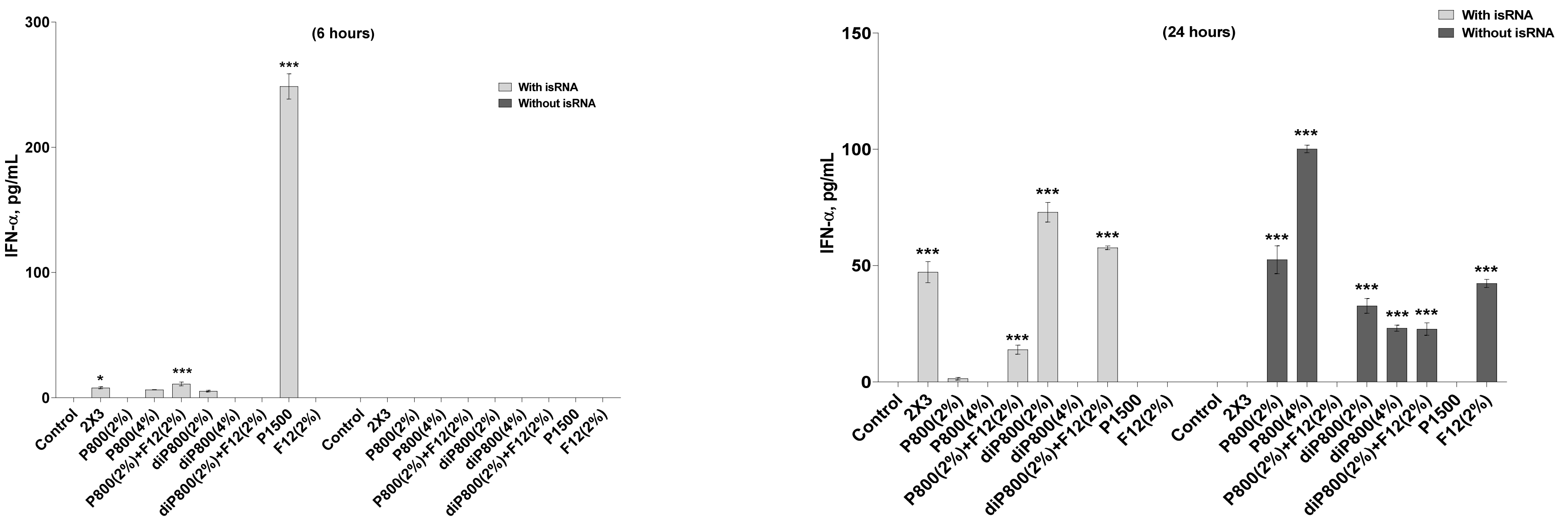
(6 hours)
IFN-α, pg/mL
300
200
100
0
With isRNA
Without isRNA
*
***
***
Control
2X3
P800(2%)
P800(4%)
P800(2%)+F12(2%)
diP800(2%)
diP800(4%)
diP800(2%)+F12(2%)
P1500
F12(2%)
(24 hours)
150
100
50
0
***

B

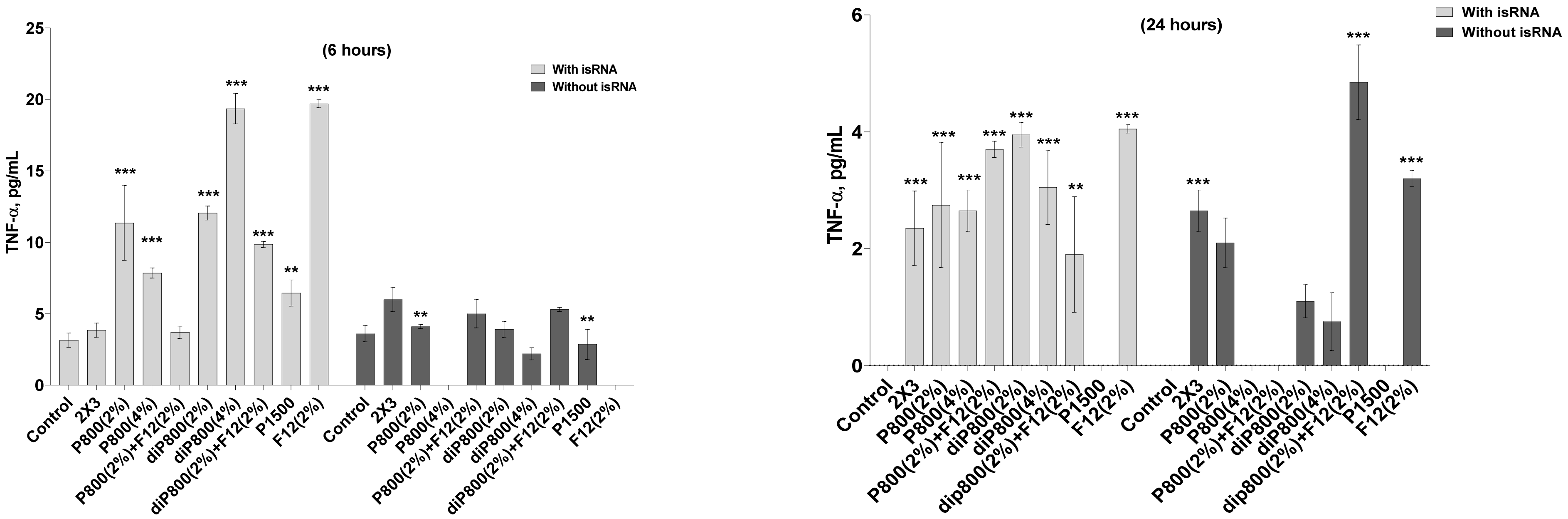
(6 hours)
TNF-α, pg/mL
25
20
15
10
5
0
With isRNA
Without isRNA
***
**
Control
2X3
P800(2%)
P800(4%)
P800(2%)+F12(2%)
diP800(2%)
diP800(4%)
diP800(2%)+F12(2%)
P1500
F12(2%)
(24 hours)
6
4
2
0
dip800(2%)
dip800(4%)
dip800(2%)+F12(2%)

C

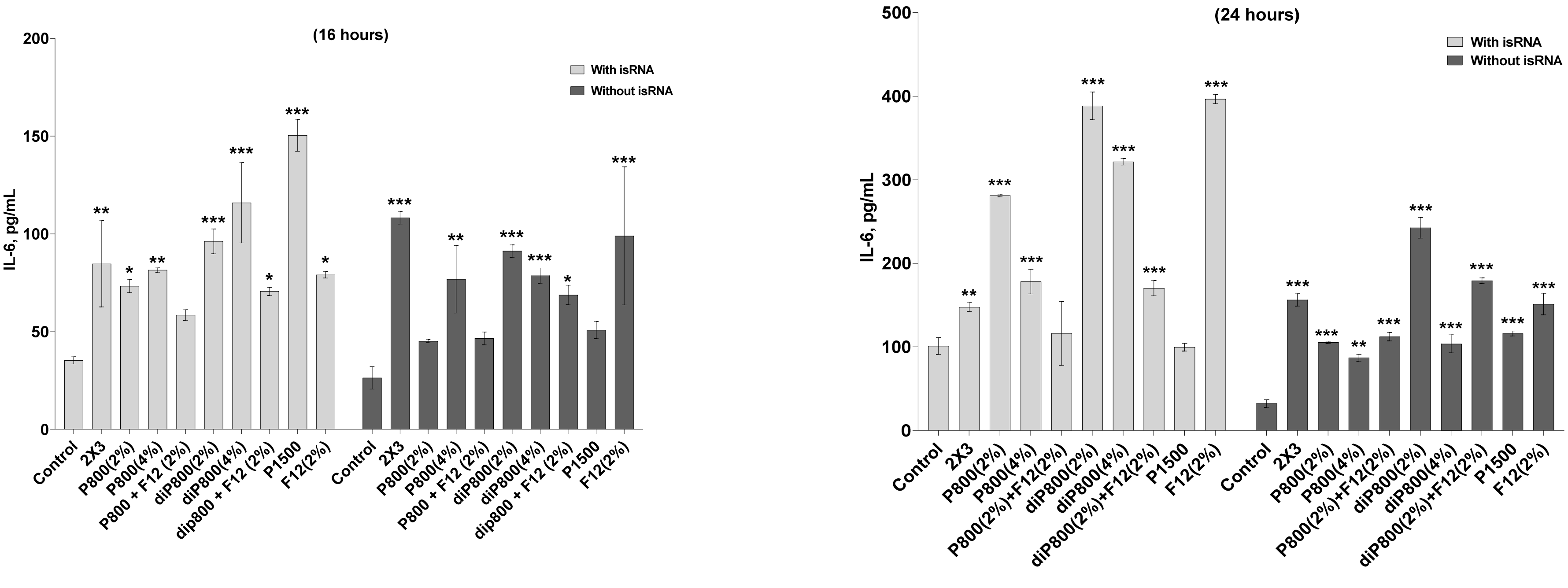

Supplementary Figure S1. Cytokines levels in human PBMCs primary cell culture after the transfection of isRNA/liposomes. The levels of IFN-α **(A)** and TNF-α **(B)** 6 and 24 h after transfection, the levels IL-6 **(C)** 16 and 24 h after transfection were measured by ELISA. The data represent mean ± standard deviation (SD). Statistically significant differences between samples treated with isRNA/liposomes and the samples treated with only liposomes are indicated by asterisk (* = P value <0.05, ** = P value<0.01, *** = P value <0.001); ordinary two-way ANOVA, Dunnett's multiple comparisons test.

RLS40

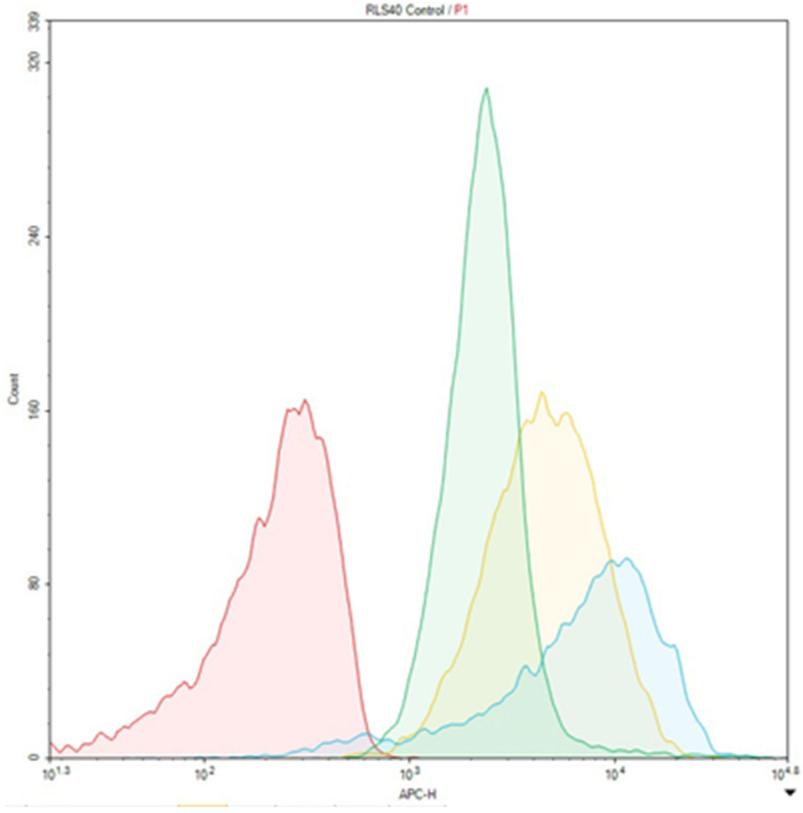

B16

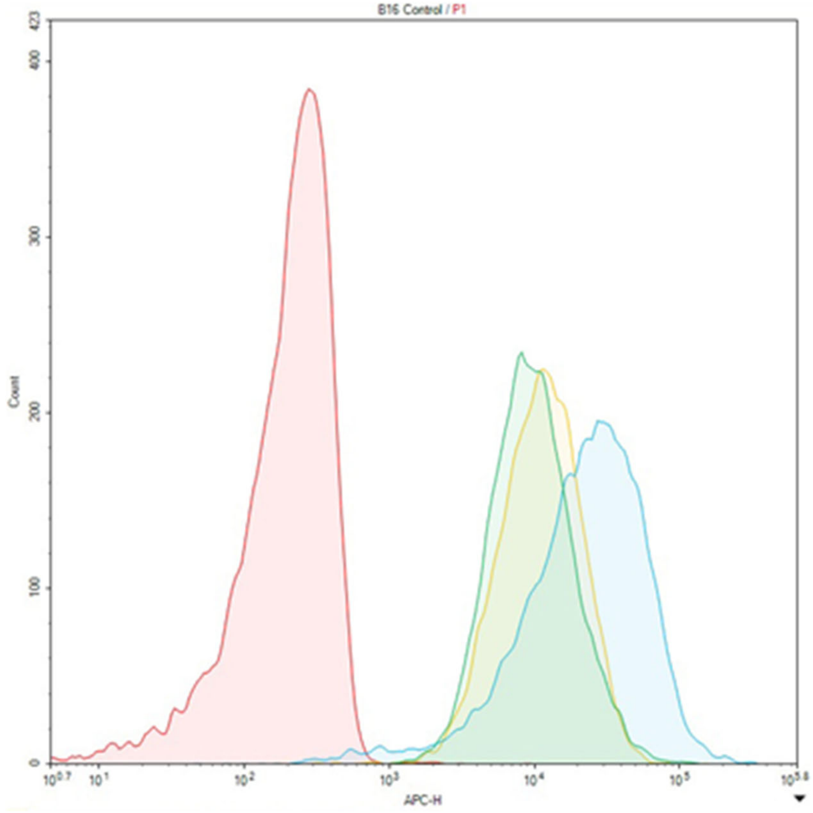

Supplementary Figure S2. Representative flow histograms of Cy5.5-isRNA and its complexes with liposomes accumulation in B16 and RLS40 cells. The accumulation was measured 4 h after transfection of Cy5.5-isRNA mediated by liposomes. Red – untreated cells cell, Green – cells incubated with -siRNA, Yellow – cells incubated with Cy5.5-isRNA/P2000 complexes, Blue – cells incubated with Cy5.5-isRNA/2X3-DOPE complexes.
